# Molecular evolution of the LNX gene family

**DOI:** 10.1186/1471-2148-11-235

**Published:** 2011-08-09

**Authors:** Michael Flynn, Orthis Saha, Paul Young

**Affiliations:** 1 Department of Biochemistry, University College Cork, Cork, Ireland

**Keywords:** ligand of numb protein X, LNX, PDZRN3, PDZRN4, PDZK4, MUPP1, INADL, LNX3 homology domain, LNX3H domain

## Abstract

**Background:**

LNX (Ligand of Numb Protein-X) proteins typically contain an amino-terminal RING domain adjacent to either two or four PDZ domains - a domain architecture that is unique to the LNX family. LNX proteins function as E3 ubiquitin ligases and their domain organisation suggests that their ubiquitin ligase activity may be targeted to specific substrates or subcellular locations by PDZ domain-mediated interactions. Indeed, numerous interaction partners for LNX proteins have been identified, but the *in vivo *functions of most family members remain largely unclear.

**Results:**

To gain insights into their function we examined the phylogenetic origins and evolution of the *LNX *gene family. We find that a *LNX1/LNX2*-like gene arose in an early metazoan lineage by gene duplication and fusion events that combined a RING domain with four PDZ domains. These PDZ domains are closely related to the four carboxy-terminal domains from multiple PDZ domain containing protein-1 (MUPP1). Duplication of the *LNX1/LNX2*-like gene and subsequent loss of PDZ domains appears to have generated a gene encoding a LNX3/LNX4-like protein, with just two PDZ domains. This protein has novel carboxy-terminal sequences that include a potential modular LNX3 homology domain. The two ancestral *LNX *genes are present in some, but not all, invertebrate lineages. They were, however, maintained in the vertebrate lineage, with further duplication events giving rise to five LNX family members in most mammals. In addition, we identify novel interactions of LNX1 and LNX2 with three known MUPP1 ligands using yeast two-hybrid asssays. This demonstrates conservation of binding specificity between LNX and MUPP1 PDZ domains.

**Conclusions:**

The *LNX *gene family has an early metazoan origin with a LNX1/LNX2-like protein likely giving rise to a LNX3/LNX4-like protein through the loss of PDZ domains. The absence of LNX orthologs in some lineages indicates that LNX proteins are not essential in invertebrates. In contrast, the maintenance of both ancestral *LNX *genes in the vertebrate lineage suggests the acquisition of essential vertebrate specific functions. The revelation that the LNX PDZ domains are phylogenetically related to domains in MUPP1, and have common binding specificities, suggests that LNX and MUPP1 may have similarities in their cellular functions.

## Background

**Figure 1:**
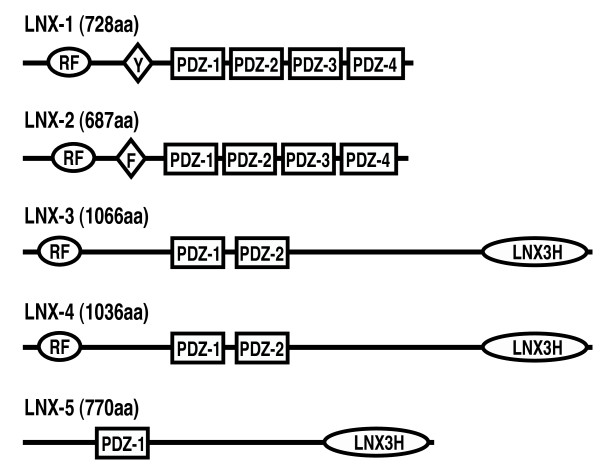
**Domain structures of mammalian LNX proteins**. LNX1 and LNX2 share an identical domain structure, as do LNX3 and LNX4. LNX5 resembles LNX3 and 4 but lacks the RING and one PDZ domain. The regions of LNX3, 4 and 5 carboxy-terminal to the PDZ domains show significant sequence similarity, particularly in the last 150 amino acids that we define here as the LNX3 homology (LNX3H) domain. The number of amino acids (aa) in the murine LNX proteins are indicated. Isoforms of LNX1, 3 and 4 resulting from alternative splicing and alternative promoter usage are not shown. RF = RING finger domain; PDZ = PDZ domain; Y = NPXY motif; F = NPXY motif.

In most mammals the LNX (Ligand of Numb Protein-X) or PDZRN (PDZ and RING) family of proteins consists of five members that, in the interest of clarity, we shall refer to hereafter as LNX1 - LNX5. These proteins are characterized by the presence of a RING domain (Really Interesting New Gene) followed by between one and four PDZ domains (PSD-95, DlgA, ZO-1). Within the LNX family, LNX1 and LNX2 are closely related and share an identical domain structure consisting of one RING and four PDZ domains (Figure 1)[1]. LNX3 and LNX4 (PDZRN3 and PDZRN4) typically have a RING domain and two PDZ domains; they are very similar to each other but more distantly related to LNX1 and LNX2 [2]. Although LNX5 (PDZK4, PDZRN4L) lacks the RING domain, it is clearly a member of the family based on high sequence homology to LNX3 and LNX4 [3,4]. Within vertebrates, the presence of RING and PDZ domains in one protein is unique to the LNX family. RING domains function as the catalytic component of E3 ubiquitin ligases - enzymes that catalyze the final step in the attachment of ubiquitin to substrate proteins and are believed to confer specificity to the ubiquitination process [5]. PDZ domains are an important and abundant class of protein-protein interaction domains that often bind to the carboxy-terminus of their ligands [6,7]. The combination of RING and PDZ domains in the LNX family proteins suggests that the ubiquitin ligase activity of these proteins may be targeted to specific substrate proteins by PDZ domain-mediated interactions.

LNX1 was identified as a protein that interacts with Numb and named Ligand of Numb protein X [8]. Numb is a component of the Notch signaling pathway that functions in the specification of cell fates during development and is known to control cell numbers during neurogenesis in vertebrates [9]. LNX1 can ubiquitinate Numb, thereby targeting Numb to proteasomal degradation [8,10]. The Numb-LNX interaction involves a four amino acid NPAY motif in LNX1 binding to a phosphotyrosine-binding domain in Numb [8,10,11]. Subsequent to the characterisation of LNX1, the highly homologous LNX2 protein was identified and shown to interact via its NPAF motif with Numb, as well as with its paralog Numblike [1]. While the ability of LNX1, and likely LNX2, to ubiquitinate Numb and thereby modulate Notch signaling in cultured cells is very clear [10,11], this function has not yet been demonstrated for LNX proteins *in vivo *during development.

Apart from Numb, several other interaction partners of LNX1 and LNX2 have been identified. These include signaling proteins such as the ErbB2, RhoC and c-Src, the presynaptic protein CAST, the melanoma/cancer-testis antigen MAGEB18 and several proteins associated with cell junctions such as claudin-1, JAM4 and the Coxsackievirus and adenovirus receptor (CAR)[12-19]. Of these only c-Src and claudin-1 were shown to be LNX1 substrates [16,17]. Ubiquitination by LNX1 targets c-Src for proteasomal degradation, whereas claudin-1 is endocytosed and trafficked to lysosomes [16,17]. The interactions with claudin-1, JAM4 and CAR suggest a possible role for LNX in the formation or remodeling of cell-cell junctions. In agreement with this, ectopic expression of LNX1 can remove claudins from cell junctions and can enhance TGFβ-induced epithelial to mesenchymal transition in epithelial cells [16,20]. However, the *in vivo *relevance of any of the aforementioned LNX1 or LNX2 interactions has yet to be demonstrated definitively. This is probably due to the fact that LNX1 and LNX2 proteins are expressed at exceedingly low levels in cell lines and *in vivo*[18,20]. Despite widespread mRNA distribution [1], reports on LNX1 or LNX2 protein expression are limited. LNX1 protein was found to localize very specifically within perisynaptic Schwann cells at neuromuscular junctions [18]. Both LNX1 and LNX2 proteins are present in spermatozoa, and LNX2 expression was reported in a subset of blood vessels during embryonic development [18,21,22]. However, the *in vivo *function of LNX1 and LNX2 proteins, in either these or other contexts, remain largely uncertain.

LNX3 (PDZRN3, SEMCAP3) was first identified based on its interaction in a yeast two-hybrid screen with a Semaphorin (Sema4C)[23]. A similar approach identified PDZ domain-mediated interactions of LNX3 with neuroligin1, ephrin-B2 and the GluR2 glutamate receptor subunit, but none of these interactions have been characterized further [24]. It was Ko *et al *(2006) who provided the first detailed description of LNX3 function [25]. They reported that LNX3 mRNA is most highly expressed in heart and skeletal muscle and that LNX3 was required for the differentiation of C2C12 myoblasts into myotubes. The same group subsequently described a role for LNX3 in the BMP-2 -induced differentiation of C2C12 cells into osteoblasts [26]. In this context LNX3 acts as a negative regulator of osteoblast differentiation by inhibiting Wnt-β-catenin signaling. A distinct role for LNX3 in neuromuscular junction formation has also been reported [27]. Here LNX3 was found to interact with and ubiquitinate the muscle specific tyrosine kinase (MuSK), thereby promoting its endocytosis and negatively regulating the cell surface expression of this key regulator of postsynaptic assembly. In agreement with this, transgenic overexpression of LNX3 was found to perturb neuromuscular junction formation *in vivo*[27]. Overall LNX3 appears to play distinct roles in the development and differentiation of both muscle and osteoblasts and is the best characterized of the mammalian LNX proteins.

LNX4 (PDZRN4/PDZRN4S) and LNX5 (PDZRN4L/PDZK4) were identified *in silico *based on homology to LNX3 [2,3]. LNX4 is almost completely uncharacterized apart from a report that it is down regulated in the hypothalamus of Sim1 knockout mice [28]. LNX5 was identified as a gene up-regulated in synovial sarcomas and was found to promote proliferation of synovial sarcoma cells [4]. In normal tissues LNX5 mRNA expression is largely confined to the brain, but its physiological function here is unknown [4].

In the present study, we take advantage of the availability of complete genome sequences for diverse metazoan species to examine the evolution of the *LNX *gene family for the first time. In particular, we focus on the phylogenetic origins of the family and the evolutionary relationship between LNX1/2-like and LNX3/4-like proteins. Our analysis traces the ancestral *LNX *gene to the earliest metazoan lineage. We characterize what may be a novel, modular protein domain present in LNX3, -4 and -5. We also reveal a close phylogenetic relationship between the LNX and MUPP1 protein families. Based on this relationship, we identify novel LNX1- and LNX2-interacting proteins.

## Results

### Identification of LNX1/LNX2 orthologs

LNX1 and LNX2 share exactly the same domain architecture (Figure 1) and display 50% amino acid sequence identity. Orthologs of both *LNX1 *and *LNX2 *are found in virtually all vertebrates for which complete genome sequences are available. In addition, fish, marsupial, amphibian and bird genomes have a third *LNX1/2*-like gene that has been termed *LNX-like *or *LNX2b *in zebra fish [29,30] and confusingly called *LNX3 *in chicken and marsupials [31]. We suggest that this paralog be called *LNX2b *to reflect the fact that it encodes a protein that is most similar to LNX2. Eutherian mammals have only two *LNX1/2*-like genes with the loss of *LNX2b *occuring when it underwent pseudogenization and contributed several exons to the non-coding *Xist *RNA that controls X-chromosome inactivation [31].

**Figure 2:**
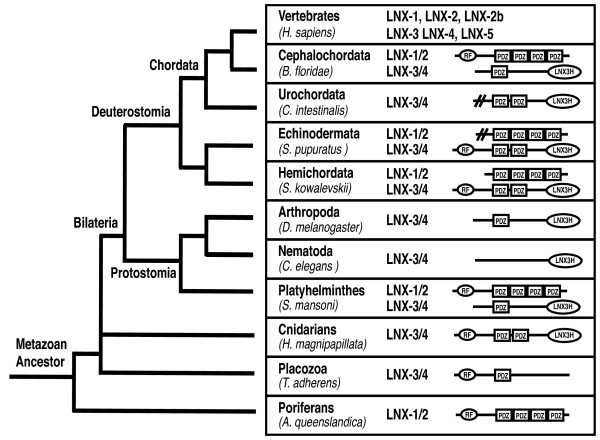
**Identification of invertebrate LNX orthologs**. Lineages in which LNX orthologs can be identified are represented on a simplified phylogenetic tree of metazoans. Domain structures of predicted proteins from a representative species in each lineage are indicated. Proteins are classified as being orthologs of vertebrate LNX1 and LNX2 (LNX1/2) or vertebrate LNX3 and LNX4 (LNX3/4). LNX3/4 orthologs were identified based on the presence of both a RING and PDZ domain, or the presence of the LNX3H domain. The *C. intestinalis *and *S. pupuratus *LNX3/4 sequences are incomplete.

Database searches for invertebrate *LNX1 *or *LNX2 *orthologs identified a single *LNX *gene, most similar to *LNX2*, in representative species of some, but not all non-vertebrate bilaterian lineages (Figure 2). Thus a clear *LNX1/2 *ortholog was identified in the cephalochordate *Branchiostoma floridae *and in the platyhelminth *Schistosoma mansoni*, but not in the urochordates, arthropods, nematodes or molluscs (represented by *Ciona intestinalis, Drosophila melanogaster, Caenorhabditis elegans and Aplysia californica*). LNX1/2-related proteins that lack the RING domain were identified in the hemichordate *Saccoglossus kowalevskii *and the echinoderm *Strongylocentrotus pupuratus*. Examining more basal metazoan lineages, we did not find *LNX1/2 *orthologs in cnidaria or placozoa, but a *LNX1/2 *ortholog is found in the poriferan *Amphimedon queenslandica*[32,33]. No *LNX1/2 *ortholog was found in choanoflagellates or any other non-metazoan species. Thus the ancestral *LNX1/2*-like gene appears to have an early metazoan origin prior to the divergence of porifera (sponges) from other metazoans, but has subsequently been lost from several lineages. This gene is likely to have been duplicated in the vertebrate ancestor, giving rise to vertebrate *LNX1, LNX2 and LNX2b*.

### LNX1/LNX2 and Multiple PDZ Protein1 (MUPP1/MPP1/MPDZ1) share a common ancestor

**Figure 3:**
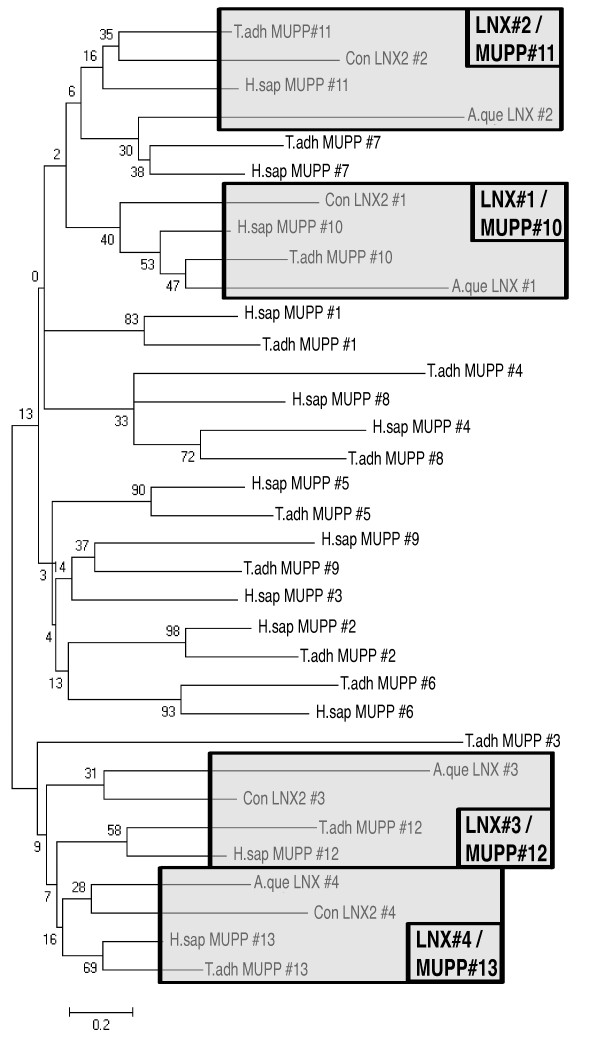
**Evolutionary relationship of the PDZ domains from LNX2 and MUPP1**. LNX2 PDZ domain sequences from *A. queenslandica *and a consensus vertebrate sequence, as well as PDZ domains from *H. sapiens *and *T. adhaerens *MUPP1 protein were analyzed. For each protein PDZ domains are numbered consecutively (#1, #2 etc). Using the Neighbor-Joining method, a bootstrap consensus tree was inferred from 1000 replicates and is taken to represent the evolutionary history of the sequences analyzed. The percentage of replicate trees in which the associated domains clustered together in the bootstrap test is shown next to the branches. The tree is drawn to scale, with branch lengths in the same units as those of the evolutionary distances used to infer the phylogenetic tree. Clustering of the four LNX PDZ domains with the last four domains of MUPP1 is highlighted in the shaded boxes. Con = a consensus vertebrate LNX2 sequence.

In searching for LNX1/2 orthologs we noticed a high degree of sequence similarity between the LNX1/2 PDZ domains and the carboxy-terminal PDZ domains from MUPP1 and its paralog INADL/PATJ. MUPP1 contains an amino terminal L27 domain and 13 PDZ domains, while INADL/PATJ has the same architecture but has just 10 PDZ domains. To examine this relationship more closely, we generated a phylogenetic tree to compare the vertebrate LNX2 PDZ domains with all the PDZ domains from human and *T. adhaerens *MUPP1 proteins as well as those from *A. queenslandica *LNX2 (Figure 3). The tree reveals a close relationship between PDZ domains 1-4 of LNX2 and domains 10-13 respectively of MUPP1. Examination of splice junctions within the PDZ domains confirmed this relationship with domains 10, 11 and12 of MUPP1 having splice junctions at exactly the same positions as domains 1, 2 and 3 respectively of LNX2 (Additional File 1). This relationship suggests that the LNX1/2 PDZ domains and the four carboxy-terminal domains of MUPP1 are derived from a common ancestor. The choanoflagellate, *Monsiga brevicollis*, lacks a LNX1/2 ortholog but has a MUPP1-like protein with 6 PDZ domains. MUPP1 and LNX2 proteins are thus likely to be derived from a MUPP1-like protein in the ancestral metazoan lineage.

### Identification of LNX3/LNX4/LNX5 orthologs

LNX3 and LNX4 proteins show close to 60% amino acid identity. The sequence similarity spans the amino terminal RING and two PDZ domains, as well as extensive regions in the non-modular carboxy-terminal half of the protein. Although LNX5 lacks a RING domain and has only one PDZ domain, it is 50% identical to LNX3 and LNX4 and contains the conserved carboxy-terminal regions, confirming it as a true paralog. Most vertebrate genomes encode both a *LNX3 *and *LNX4 *ortholog, though a few species lack one or the other gene (but not both). Specifically, *Canis familiaris *(dog) and *Ornithorhynchus anatinus *(platypus) appear to lack *LNX3*, while no *LNX4 *homolog is found in *Monodelphis domestica *(opossum). *LNX5 *is found in most amphibian, fish and mammalian species but seems to be absent from birds. Invertebrate orthologs of LNX3/LNX4 containing RING and PDZ domains are found in some metazoan lineages including hemichordates (*S. kowalevskii) and *echinoderms(*S. pupuratus)*, cnidarians (*Hydra magnipapillata) *and placozoans *(Trichoplax adhaerens) *(Figure 2). All of these invertebrate LNX3 orthologs contain two PDZ domains with the exception of *T. adhaerens *in which the second PDZ is not discernible. No PDZ domain-containing LNX3 homolog is found in the draft assembly of the poriferan *A. queenslandica *genome [33], nor was an ortholog found in the choanoflagellate *M brevicolli*s. A LNX3/4-like protein can thus be traced back to the common ancestor of cnidarians, placozoans and bilaterians. Duplications of the ancestral *LNX3/4-*like gene gave rise to *LNX3, LNX4 and LNX5 *in vertebrates with LNX5 losing the exons encoding the RING and one PDZ domain.

### Characterisation of a LNX3 homology domain

**Figure 4:**
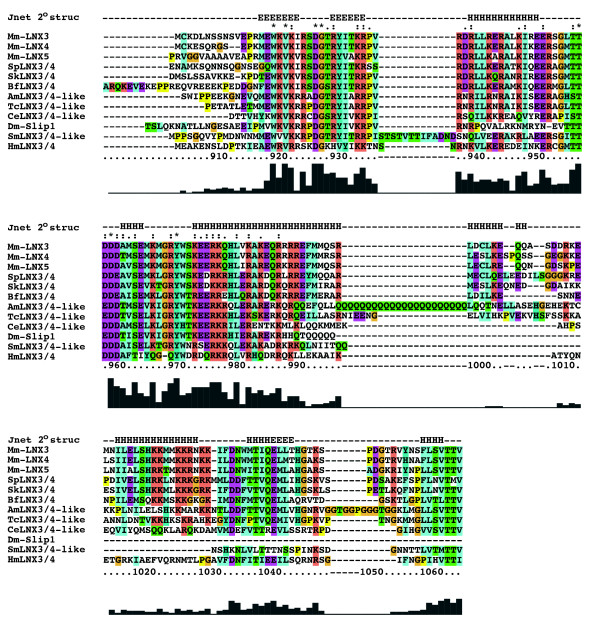
**Multiple sequence alignment of the LNX3 homology domain**. An alignment prepared using ClustalX of the last 150 amino acids of murine LNX3, LNX4 and LNX5 as well as LNX3/4 orthologs from diverse invertebrate species. Sequence conservation is plotted beneath the alignment and conserved residues marked and color coded according to the default CustalX settings. A Jnet secondary structure prediction for LNX3 was generated using the alignment and is shown on top (E = extended or beta sheet conformation, H = helical). Amino acids numbers for mouse LNX3 are indicated. Mm *= Mus musculus; *Sp *= S. pupuratus; *Sk *= S. kowalevskii; *Bf *= B. floridae; *Am *= Apis mellifera; *Tc *= Tribolium castaneum; *Ce *= C. elegans; *Dm *= D. melanogaster; *Sm *= S. mansoni; *Hm *= H. magnipapillata*

The carboxy-terminal half of LNX3 lacks any identifiable domains annotated in the Pfam, SMART or InterPro databases [34-36], yet this region is highly conserved in LNX4 and LNX5 and in all invertebrate LNX3/4 orthologs with the exception of *T. adhaerens*. Within this region, the last 150 amino acids are particularly similar and the degree of cross-species sequence identity is as high as that for the PDZ and RING domains. In fact, many invertebrate lineages have LNX3/4-like proteins that contain this highly conserved carboxy-terminal region, but lack the characteristic RING domain and may also be missing one or both of the PDZ domains (Figure 2). Lineages that have such "RING-less" or "RING and PDZ-less" LNX3/4 relatives include arthropods (Slip1 in *D. melanogaster*), nematodes (*C. elegans*) and cephalochordates (*B. floridae*). Katoh and Katoh (2004) had previously identified conserved sequences shared by LNX3/4 and *D. melanogaster *Slip1 (CG1783). With the availability of sequences from other species, we can better define this conserved region that we term the LNX3 homology (LNX3H) domain. Figure 4 shows a multiple sequence alignment of the carboxy-terminal regions of vertebrate LNX3, 4 and 5 and selected invertebrate LNX3 orthologs. The high degree of sequence conservation between diverse species within this region is apparent and indeed automated domain detection methods identify this region as a potential domain (Prodomain family PD140124, ADDA family 37421)[37,38]. Such conservation is not seen in the rest of the sequence (full alignment available in Additional file 2). Notably the *D. melanogaster *Slip1 is shorter than all other sequences examined including several other arthropods, thus the consensus LNX3 homology region is more extensive than that previously identified [3].

We used the multiple sequence alignment to generate a protein secondary structure prediction for the LNX3H domain on the Jpred 3 server [39]. The Jpred algorithim predicts several alpha-helical regions with a high degree of confidence as well as some potential beta sheets or regions with an extended confirmation (Figure 4). The conservation of this region across diverse species and secondary structure prediction are consistent with this 150 amino acid region being a modular folded domain with functions independent of the RING and PDZ domains.

### Phylogenetic relationship between LNX1/2 and LNX3/4

The combination of an amino terminal RING domain with carboxy-terminal PDZ domains (RING-PDZ_n_) appears to be unique to the LNX family. In principle, however, the common domain architecture of LNX1/2-like and LNX3/4-like proteins may have arisen independently. PDZ and RING domains are abundant in metazoan genomes and so the independent evolution of a RING-PDZ_n _architecture more than once is plausible. Indeed, in arthropods this domain combination does seem to have arisen independently, but in the reverse order (PDZ-RING). Since LNX1/2 and LNX3/4 have only limited sequence homology in non-modular regions we examined the relationships between the RING and PDZ domains of LNX1-4. Using a BLASTP search, we find that the most similar protein sequences to the RING domain of human LNX1 are the RING domains of LNX2, LNX4 and LNX3 respectively (79%, 46% and 45% identity). To examine relationships between the PDZ domains, we have generated a phylogenetic tree from an alignment of all human PDZ domains (Additional file 3). We find that PDZ2 of LNX1 and LNX2 clusters very tightly with PDZ2 of LNX3 and LNX4, while the PDZ1 domains from all four proteins are likewise closely related. These close relationships between both the RING and PDZ domains of LNX1/2 and LNX3/4 as compared to any other sequences in the human genome argue strongly that they did indeed arise from a single evolutionary event that combined an amino-terminal RING domain with PDZ domains at the carboxy-terminal side.

**Figure 5:**
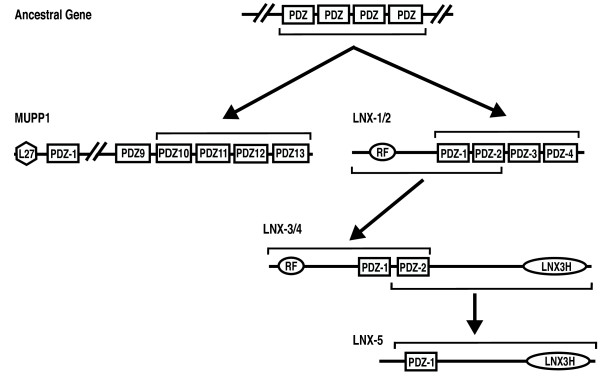
**A model of the molecular phylogeny of the LNX family**. A LNX1/2**-**like protein is likely to have arisen through genomic rearrangements in a very early metazoan that combined a RING domain with four PDZ domains. The LNX PDZ domains are derived from PDZ domains encoded by an ancestral gene that also gave rise to the four carboxy-terminal PDZ domains of MUPP1 (PDZ 10-13). LNX3/4-like proteins resulted from the loss of two PDZ domains and the acquisition or evolution of a novel carboxy-terminal region including what we define here as a LNX3 homology domain. Gene duplications of both of these invertebrate LNX genes gave rise to the LNX1/LNX2/LNX2b and LNX3/LNX4/LNX5 subfamilies in vertebrates. A loss of exons encoding the RING and one PDZ domain occurred to give rise to LNX5.

Our analysis indicates that LNX1/2 and LNX3/4 did share a common ancestor and that the two PDZ domains in LNX3/4 correspond to PDZ domains 1 and 2 of LNX1/2. This suggests two possible scenarios for the evolution of the LNX family: 1) a LNX1/2-like protein with four PDZ domains might have arisen from a LNX3/4 like ancestor through acquisition/duplication of PDZ domains; 2) a LNX3/4-like protein with two PDZ domain may have arisen from a LNX1/2-like ancestor protein through loss of two PDZ domains. The poriferan *A. queenslandica *has a LNX2 ortholog with four PDZ domains. The most basal lineages with a clear LNX3/4 homolog are placozoans and cnidarians. While the *A. queenslandica *genome is not completely assembled, it appears that a LNX1/2-like protein predates the evolution of LNX3/4. Our analysis of LNX2 PDZ domains indicates that they are closely related to the most carboxy-terminal PDZ domains of MUPP1 and are derived from a common ancestor (Figure 3). *A. queenslandica *has a MUPP1 ortholog indicating that LNX2 and MUPP derived from a common multiple PDZ domain-containing protein in the ancestor of metazoans. We propose a model for the evolution of the LNX family in which an ancestral LNX2-like protein in the earliest metazoans gave rise to a LNX3/4-like protein with two PDZ domains (Figure 5). The highly conserved LNX3H domain is found in most lineages from cnidarians onward suggesting that it arose in LNX3/4 subsequent to the loss of the carboxy-terminal PDZ domains. LNX2 was lost in many metazoan lineages but retained in vertebrates. LNX3/4 orthologs are found in most metazoans; but in many lineages these orthologs have lost the RING domain and in some cases the PDZ domains, leaving the LNX3H domain as the most defining characteristic of this branch of the LNX family. In vertebrates, duplications of the ancestral *LNX1/2-like *gene gave rise to *LNX1, LNX2 and LNX2b *and likewise gene duplication generated *LNX3, LNX4 and LNX5 *from the *LNX3/4-like *ancestral gene.

### Identification of common LNX1/2 and MUPP1 interaction partners

The shared phylogenetic origins of the LNX2 and MUPP1 PDZ domains prompted us to examine whether the two proteins share any interacting proteins. Proteins that interact with PDZ domains 10-13 of MUPP1, or the equivalent domains from INADL/PATJ (PDZ domains 8-10), were identified by searching the primary literature and protein interaction databases. Among these proteins, Claudin-1 and CAR, have been independently reported to interact with LNX1 and/or LNX2 [15,16,40,41], while four common ligands of MUPP1 and LNX1 were reported in a large-scale analysis of PDZ domain interactions using protein microarrays (Cftr, EphA7, PKC and SSTR2)[42]. We reasoned that novel ligands for LNX PDZ domains might be identified among known MUPP1 or INADL/PATJ interactors. Using the yeast two-hybrid system, we tested whether the carboxy-terminal peptides from three known MUPP1 interactors, HTR2C (Serotonin receptor 2C subunit [43]), SynGAP1 (synaptic GTPase activating protein [44]) and TAPP1 (tandem pleckstrin homology-domain containing-protein-1 [45]), could also bind to LNX1 or LNX2. In addition, we examined the previously reported LNX1 and MUPP1 ligand, EphA7 in order to confirm these interactions in an independent assay [42]. All four proteins interacted with MUPP1-PDZ10-13 as judged by activation of both the His3 and βgal reporter genes (Table 1). This serves as a positive control for the expression and functionality of the bait constructs. To test for interactions with LNX1 we used constructs lacking either the RING domain (NPAY-PDZ4) or the 3^rd ^and 4^th ^PDZ domains (RING-PDZ2) as prey. These truncated constructs were used because, in contrast to LNX2, full-length LNX1 protein was not functional in the yeast two-hybrid system. HTR2C did not show any evidence of an interaction with LNX1 constructs and showed only moderate expression of one reporter gene when binding to LNX2 was assessed (Table 1). In contrast, SynGAP1, TAPP1 and EphA7 all showed an interaction with LNX1 and/or LNX2, as judged by activation of both reporter genes. Some LNX1 interactions were only observed using the carboxy-terminal truncated RING-PDZ2 construct. This may be because PDZ1 of LNX1 can bind its own carboxy-terminus, thereby preventing other proteins from binding efficiently to this domain [1]. To demonstrate the specificity of these interactions, the ability of carboxy-terminal peptides from SynGAP1, TAPP1 and EphA7 to bind to unrelated PDZ domains was assessed. No interactions were observed with the PDZ domains of either ERBB2IP or Nos1, while only SynGAP1 showed an interaction with the PDZ domain-containing protein Dlg2 (Table 1). This interaction is expected based on the previously reported interaction of SynGAP1 with Dlg4 [46,47]. Overall these observations indicate that the interactions of SynGAP1, TAPP1 and EphA7 with LNX proteins are very specific.

**Table 1:** Interaction of LNX1 and LNX2 with known MUPP1 ligands

Prey	Empty Vector		MUPP1 (PDZ 10-13)		LNX1 (NPAY-PDZ4)		LNX1 (RING-PDZ2)		LNX2		ERBB2IP (PDZ)		Nos1 (PDZ)		Dlg2	
								
Bait	His3	β-gal	His3	β-gal	His3	β-gal	His3	β-gal	His3	β-gal	His3	β-gal	His3	β-gal	His3	β-gal
**TAPP1**	-	-	+++	+++	-	-	+++	+++	-	-	-	-	-	-	-	-
**SynGAP1**	-	-	+++	+++	++	-	+++	+++	+++	+++	-	-	-	-	+++	+++
**EphA7**	-	-	+++	++	+++	+	+++	+++	+++	+++	-	-	-	-	-	-
**HTR2C**	-	-	+++	+++	-	-	-	-	++	-	n/d	n/d	n/d	n/d	n/d	n/d

The ability of carboxyl terminal sequences from the indicated MUPP1 interacting proteins (baits) to interact with LNX1 and LNX2 (preys) was tested using the yeast two-hybrid system. An empty prey vector and a MUPP1 prey were included as negative and positive controls respectively. Interactions with unrelated PDZ domain-containing proteins (ERBB2IP, Nos1 and Dlg2) were assessed to demonstrate the specificity of binding to LNX proteins. Interactions between bait and prey were indicated by expression of two reporter genes - *His3 *and *LacZ *coding for β-galactosidase (β-gal). Truncated LNX1 constructs were used because the full-length protein was not functional in the yeast two-hybrid system. n/d = not determined.

Our strategy of screening known MUPP1 interacting proteins was thus successful in identifying three novel ligands for the LNX1 and LNX2 PDZ domains. We also confirmed the common interaction of LNX1, LNX2 and MUPP1 with EphA7 and show that EphA7 can bind, not only to an isolated LNX1 PDZ domain [42], but also to full-length LNX2 and near full-length LNX1 proteins. To evaluate the scope for other shared interactions, we examined some of the key ligand-binding residues within the PDZ domains of LNX1, LNX2 and MUPP1 [48]. For the most part these residues are highly conserved in all three proteins (Additional file 4). In agreement with this, a recent computational analysis of PDZ domain interactions [49] predicts numerous common ligands for MUPP1-PDZ10-13 and the LNX1/LNX2 PDZ domains (Additional file 5). Many of these interactions are mediated by equivalent PDZ domains from both proteins - in agreement with their common phylogenetic origins.

## Discussion

We have undertaken the current phylogenetic analysis with a view to gleaning some insights into the functions of the poorly characterized LNX protein family. We find that the sponge *A. queenslandica *has a *LNX1/2 *ortholog. Thus the ancestral *LNX1/2 *gene would have evolved in very primitive metazoans, that likely consisted of layers of cells but lacked distinct tissues. This suggests that the original LNX1/2 protein probably had a general cellular function, one that perhaps related to the development of multicellular metazoans but was not specific to a particular tissue type or organ system. It remains to be seen whether the function of the LNX1/2 ortholog in present day poriferans is closely related to its ancestral function and whether studying LNX1/2 in poriferans could provide clues as to the roles of LNX1/2 orthologs in other lineages.

Whatever the role of the ancestral LNX1/2 protein was, it is apparent that this function is not essential in invertebrates since many lineages, such as arthropods and nematodes, lack a LNX1/2-like protein. Nevertheless the *LNX1/2 *gene was maintained in the lineage leading to vertebrates and duplicated to give rise to the paralogous *LNX1, LNX2 and LNX2b *genes in vertebrates. The duplication of the ancestral *LNX1/2 *gene was probably a result of the two rounds (2R hypothesis) of large-scale gene or whole-genome duplication that are thought to have occurred early in the vertebrate lineage [50,51]. The loss of *LNX-2b *through pseudogenization in eutherian mammals is indicative of some redundancy of function between the *LNX1/2 *paralogs, but the maintenance of at least two paralogs in all vertebrates is suggestive of an essential function for LNX1/2-like proteins in vertebrates [31]. LNX1 was first described as a ligand of Numb - an interaction that is mediated by an NPAY motif in LNX1 and the phosphotyrosine binding domain of Numb [8,11]. LNX2 has an NPAF motif that can also bind Numb [1]. It is noteworthy that the invertebrate LNX1/2 orthologs lack such a motif; therefore, they would not be expected to bind Numb or modulate Notch signaling via degradation of Numb. It is possible that the ability to interact with and regulate Numb is an essential vertebrate specific function that explains the presence of LNX1/2 orthologs in all vertebrates. This remains speculative however until the *in vivo *significance of this interaction is clarified by loss of function studies. During zebra fish embryogenesis, LNX-2b functions in the establishment of the dorso-ventral axis by modulating the activity of the transcriptional repressor Bozozok [29,30]. Whether LNX-2b in amphibians or birds, or LNX2 in mammals, has a similar function is unclear, since there does not appear to be a clear Bozozok homolog in these lineages.

Phylogenetic analysis reveals that the original LNX1/2 protein shared a common ancestor with MUPP1 and INADL/PATJ - proteins that are found at tight junctions in epithelial cells [41,52]. LNX1 has been shown to interact with the cell junction associated proteins JAM4, claudin-1 and CAR and shares the latter two interactions with MUPP1 [14-16]. Taken together these observations, along with the evolution of the ancestral LNX1/2 gene in early multicellular metazoans, support a possible function for LNX1/2 at cell-cell junctions. While endogenous LNX1 and LNX2 have not been localized to cell junctions, roles for LNX1 in the modulation of cell junctions have been suggested from studies using exogenously expressed proteins [16,20]. It is possible that low expression levels and a transient association with cell junction proteins might preclude the detection of endogenous LNX proteins at these structures. Interestingly, endogenous LNX2 and MUPP1 have been localized to the acrosomal region of mammalian spermatozoa where CAR is also found [21,53]. This is suggestive of related functions for LNX2 and MUPP1 in this context.

In addition to common interactions with claudin-1 and CAR, we have identified two known MUPP1 ligands, TAPP1 and SynGAP1 as novel interaction partners for LNX1and/or LNX2. Furthermore, we have confirmed a common interaction of MUPP1 and LNX1/LNX2 with EphA7. Full length LNX2 shows convincing interactions with EphA7 and SynGAP, while full length LNX1 interacts with EphA7. By contrast strong evidence for interactions of TAPP1 and SynGAP with LNX1 is only observed using the carboxy-terminal truncated RING-PDZ2 construct. This may be because PDZ1 of LNX1 can bind its own carboxy-terminus, thereby preventing other proteins from binding efficiently to this domain [1]. While LNX2 is also thought to bind its own C terminus in the same way as LNX1 [1], it may be that in the case of LNX2 the affinity of this interaction is lower than the affinity for SynGAP1 and TAPP1. The binding of LNX1/LNX2 to their own carboxy-termini might serve to regulate their interactions with such ligands.

TAPP1 is a phosphatidylinositol (3,4) bisphosphate binding protein that appears to function as a negative regulator of phosphoinositide 3-kinase signaling [54]. SynGAP1 is a synaptic Ras-GTPase activating protein that regulates synapse development and synaptic plasticity [55,56]. EphA7 functions in axon guidance and in the regulation of apoptosis in neural progenitors [57,58]. Confirmation and further investigation of these interactions may reveal novel roles for LNX1 and LNX2 in cell signaling and nervous system development. Overall, an appreciation of the phylogenetic relationship between the PDZ domains of LNX1/2 and MUPP1/INADL/PATJ will be important in elucidating the cellular functions of both of these poorly understood protein families.

Our model for the evolution of the LNX family indicates that an invertebrate LNX3/4 ortholog arose from the ancestral LNX1/2-like protein through loss of two PDZ domains. The most basal species with a LNX3/4 ortholog is the placozoan *T. adhaerens*. This protein, which has a RING and one PDZ domain, may represent an intermediate step in the evolution of LNX3/4. If this is the case, then the ancestral LNX1/2 would, in fact, have lost three PDZ domains and the single remaining PDZ domain might have duplicated to generate the two PDZ domains found in other LNX3/4 orthologs. Supporting this idea, an analysis of splicing junctions shows that both PDZ domains in LNX3 and in LNX4 and the single domain in LNX5 have a splice site at the same position as PDZ1 of LNX1/2 (Additional File 1). However, the relationships between these domains in a phylogenetic tree of all PDZ domains don't agree with this hypothesis (Additional File 3). Therefore, it seems equally possible that the ancestral LNX3/4 gene originally encoded two PDZ domains, one of which was subsequently lost in *T. adhaerens*. The *T. adhaerens *protein also lacks the LNX3H domain or any significant homology to other LNX3/4 orthologs in the region carboxy-terminal to the PDZ domains. Again this may represent an intermediate step in which a non modular sequence replaced some of the four LNX1/2 PDZ domains prior to the evolution of the LNX3H domain and other conserved regions.

During evolution rearrangements of existing protein domains give rise to novel domain combinations and architectures that facilitate functional diversification of proteins without the need to invent new protein modules [59]. Increased rates of domain rearrangements and the appearance of novel domain combinations are associated with major lineage diversification events, particularly the emergence of metazoans [60,61]. In addition, diversity of domains architectures increases with organismal complexity [61]. The evolution of the LNX family through the combination of RING and PDZ domains appears to be a part of this metazoan diversification of domain architectures. The addition of domains involved in binding to those with catalytic activity via fusion events has been noted to occur with a high frequency during domain rearrangements [62]. Thus a role for the LNX PDZ domains in targeting/modulating the ubiquitin ligase activity of a RING domain would be in keeping with a larger evolutionary trend. Our observation that a unique fusion event likely gave rise to the RING-PDZ_n _architecture is in keeping with the prevalence of fusion events during domain rearrangements, and the findings that domain additions occur most frequently at the ends of proteins and that most domain architectures have a single evolutionary origin [59,62-65]. However, the evolution of the reversed (PDZ-RING) domain arrangement in arthropods does seem to have occurred independently. While in principal this domain order could arise through circular permutation of the LNX architecture, such events are relatively rare [66,67], and the PDZ and RING domains in arthropod PDZ-RING proteins are not closely related to those in the LNX family arguing against this possibility. Our model of LNX protein evolution proposes that duplication of a LNX1/2-like (RING-PDZ_4_) ancestor gave rise to a LNX3/4-like (RING-PDZ_2_) protein. We cannot be certain of the evolutionary mechanisms underlying the loss of the two C-terminal PDZ domains and appearance of the LNX3H domain and other conserved region in LNX3/4/5. A duplication-degeneration mechanism [68] followed by *de novo *evolution of the putative LNX3H domain is one possibility, but a fusion event that inserted the LNX3 homology-encoding region from an existing gene into the ancestral *LNX *gene is also plausible. Sequencing of *LNX *homologs from additional basal metazoan lineages may resolve these possibilities.

Apart from *T. adhaerens*, all invertebrate and vertebrate LNX3/4 orthologs contain the highly conserved carboxy-terminal region of 150 amino acids that we define here as the LNX3H domain. A previous comparison of LNX3 and the *D. melanogaster *LNX3/4-like protein Slip1 had identified two conserved regions apart from the PDZ domains [2]. By analyzing a more diverse set of invertebrate species, we find that only one of these, the LNX3H domain located at the carboxy-terminal, is widely conserved. Furthermore, Slip1 is truncated relative to all other LNX3/4-like sequences examined, including those in a variety of other arthropods. The *D. melanogaster *sequence is thus anomalous and the region of highly conserved sequence in LNX3/4-like proteins is about 60 amino acids longer than previously described. Secondary structure prediction for the LNX3H region indicates a high helical content, while an examination of amino acid composition reveals a very high proportion of charged amino acids, especially positively charged lysines and arginines (25%). These observations are consistent with this region folding as a globular domain, but biophysical and structural approaches must now be applied to determine this definitively.

Cnidarians make up the most basal lineage that have the LNX3H domain, and representative organisms in all bilaterian lineages examined encode a protein in which this domain is present. The basic LNX3/4 domain architecture seen in cnidaria has been maintained in vertebrates, yet in many invertebrate lineages, the RING and in some cases the PDZ domains have been lost. The LNX3H domain is thus more conserved across diverse invertebrate lineages than the RING and PDZ domains. Some species such as *C. elegans *have a protein that contains the LNX3H domain in the absence of any other recognizable domains. Together, these observations suggest that the LNX3H domain has functions that are independent of the RING and PDZ domains and that may be more important than the functions of the other domains, at least in invertebrates. To date, however, the function of the LNX3H domain in any species is completely unclear. Since the LNX3H domain is conserved in LNX5, its presence in a protein may be a better way of defining the LNX3/4/5 subfamily than the presence of both a RING and PDZ domain.

## Conclusions

This study represents the first, thorough examination of the molecular evolution of the LNX gene family. Based on our findings an exploration of common interactions and cellular functions of LNX1/2 and MUPP1 is merited. Structural and functional characterization of the LNX3H domain should also provide insights into the LNX3/4/5 subfamily. Our analysis thus provides a useful framework for the further characterization of the enigmatic LNX proteins.

## Methods

### Retrieval and validation of sequences for LNX orthologs

Several approaches were used to retrieve a comprehensive set of both vertebrate and invertebrate LNX protein sequences. Initially, a search was carried out within the NCBI Protein Database using the following keywords as a query; "Ligand of numb protein X 1", "Ligand of numb protein X 2", "Ligand of numb protein X 3", "Ligand of numb protein X 4", "LNX1", "LNX2", "LNX3", "LNX4", "PDZRN3", "PDZRN4", "PDZD4", "SEMACAP3" and "Semaphorin cytoplasmic domain-associated protein 3". Sequences were retrieved and each of these sequences was used to query the NCBI Protein Database using BLASTp [69] to identify closely related sequences.

To complement the above approach the Conserved Domain Architecture Retrieval Tool (CDART) was also used [70]. A list of all sequences which contain both a RING and PDZ domain was generated. This list was searched for sequences that were not found during the Entrez and BLAST searches. Finally to retrieve sequences that may not be fully or correctly annotated, translated BLAST (tBLASTN) searches were performed against any assembled RefSeq genome sequences for which LNX orthologs had not been found using the previous approaches. The number of sequences retrieved using each of these approaches were as follows: keyword queries - 62 sequences, pBlast - 18 sequences, CDART -13 sequences, tBLASTN - 159 sequences. The LNX1/2 sequence from *A. queenslandica *was initially obtained from Sakarya *et al.*, who assembled sequences of proteins containing PDZ domains from EST and genomic trace data [32]. The protein sequences were downloaded from the supplemental material accompanying this paper and scrutinised to identify LNX homologs. In the latest version of the *A. queenslandica *genome the LNX1/2 sequence is identified as transcript Aqu1.226425 http://www.metazome.net/amphimedon. tBLASTN searches of this draft sequence did not reveal any LNX3/4 ortholog.

Alignments of all retrieved seqeuences were generated using ClustalX and visually inspected to verify that sequences were indeed LNX homologs and to distinguish which LNX protein each sequence represented, as many automatically generated annotations were incorrect. Domain architectures for all identified LNX homologs were determined using SMART [71]. The number of PDZ domains and presence or absence of the RING domain was noted for each sequence. Accession numbers of LNX homologs identified or referred to in this study are provided as supplemental material online (Additional file 6).

### Alignment and generation of phylogenetic trees for individual PDZ domains

Sequences of all human PDZ (PF00595) domains were retrieved from the Pfam database [72]. Duplicate sequences resulting from multiple isoforms of the same protein were removed. An alignment for all human PDZ domains was generated using MUSCLE [73,74]. A phylogenetic tree for all human PDZ domains was generated using the maximum likelihood method in PhyML [75]. The Jones-Taylor -Thornton substitution model was used [76].

A phylogenetic tree for LNX2 and MUPP1 PDZ domains from vertebrate and early metazoan was generated as follows. An alignment of the *Homo sapiens, Mus musculus, Gallus gallus, Xenopus tropicalis *and *Danio rerio *LNX2 sequences was generated using MUSCLE [73,74]. A consensus LNX2 sequence for vertebrates was derived from this alginment using Jalview [77]). Sequences of individual PDZ domains were then extracted from the vertebrate consensus sequence as well as from *A. queenslandica *LNX2 and *H. sapiens *and *T. adhaerens *MUPP1. Domain boundries were identified by searching against the PFAM database. An alignment of the extracted PDZ domains was first generated in MUSCLE [73,74]. Phylogeny was inferred using the Neighbour Joining method in MEGA4 [78,79]. 1000 bootstrap samples were chosen and the Jones-Taylor-Thronton substitution model was used [76]. The evolutionary distances are in the units of the number of amino acid substitutions per site. All positions containing gaps and missing data were eliminated from the dataset. There were a total of 69 positions in the final dataset. Graphical rendering of phylogenetic trees was performed using FigTree [80].

### Sequence analysis and secondary structure prediction for LNX3/4/5-like proteins

*M. musculus *LNX3, LNX4, LNX5 and various invertebrate LNX3 and LNX3-like orthologs were aligned using ClustalX [81]. To identify protein domains within these orthologs, both full length protein sequences as well as the conserved carboxy terminal LNX3H regions alone, were used to search the Pfam, SMART and InterPro databases [34-36]. Searches of Pfam domains were performed using an E-value cutoff of 1 and did not retrieve any significant matches in either the Pfam A or B databases for the regions carboxy terminal to the PDZ domains in LNX3/4/5 orthologs. Even raising the E-value threshold did not identify any Pfam domains in these regions. Searches of the SMART and InterPro databases were performed using default parameters and likewise did not identify domains in the carboxy terminal regions of LNX3/4/5 orthologs. To determine if the conserved carboxy terminal regions of LNX3/4/5 were identified as potential domains using automated domain detection methods, searches of the ProDom and ADDA database was performed. *M. musculus *LNX3 was used as input sequence. For ProDom searches the blastp and multiple alignment options and an expect value of 0.01 were selected. The ADDA database was searched using default parameters.

Secondary structure analysis for the LNX3H region of *M. musculus *LNX3 was performed using the Jpred3 secondary structure prediction server [39]. Secondary structure prediction was performed using the last 160 amino acids of the multiple sequence alignment described above. A second prediction performed using just LNX3 as an input sequence and an alignment generated by the Jpred server gave very similar results. The secondary structure prediction was manually plotted onto the multiple sequence alignment in Adobe Illustrator.

### Yeast two-hybrid protein interaction analysis

Oligonucleotides coding for the carboxy-terminal ten amino acids of murine HTR2C, TAPP1, SynGAP1 and EphA7 were cloned into the pLex bait vector [82]. cDNAs sequences encoding murine MUPP1 (amino acids 1600-2058), LNX1 (amino acids 132-728 or amino acids 1-491), LNX2 (amino acids 1-687), Nos1 (amino acids 2-100), rat Dlg2 (amino acids 1-851) and human ERBB2IP (amino acids 1254-1371) were amplified by polymerase chain reaction and cloned into the pGAD10 prey vector (Clontech). Bait and prey plasmids were co-transformed into the *Saccharomyces cerevisiae *L40 reporter strain and grown on selection agar plates lacking leucine and tryptophan to select for plasmids. *His3*-reporter gene activation was monitored by restreaking multiple colonies onto plates that also lacked histidine. Filter assays for β galactosidase activity were performed as described in the Clontech manual, using X-gal (5-bromo-4-chloro-3-indolyl-β-D-galactopyranoside) as color substrate. Briefly, yeast cells were transferred to nylon membranes (GE Healthcare) and frozen twice in liquid nitrogen to lyse cells. The membrane was then placed on a filter paper soaked in Z buffer (130 mM Na_2_HPO_4_/NaH_2_PO_4_, pH 7, 10 mM KCl, 1 mM MgSO_4_7H_2_0, 0.001% β mercaptoethanol, 0.85 mg/ml X-gal) and incubated for 6-12 hours at 37°C.

## Authors' contributions

MF identified and characterised LNX orthologs and generated alignments and phylogenetic trees for individual PDZ domains. OS carried out searches for MUPP1 ligands and performed yeast-two-hybrid analysis. PY conceived the project and analysed the LNX3 homology domain. MF, OS and PY wrote the paper and all authors read and approved the final manuscript.

## Supplementary Material

Additional file 1 **Analysis of splicing junctions in LNX1, LNX2, LNX3, LNX4 and MUPP1 PDZ domains**. An alignment of PDZ domain sequences with splicing junctions highlighted. Click here for file

Additional file 2 **Multiple sequence alignment of LNX3, LNX4 and LNX5 and their invertebrate orthologs**. An alignment of full length murine LNX3, LNX4 and LNX5, as well as LNX3/4 orthologs from diverse invertebrate species. Click here for file

Additional file 3 **Phylogenetic tree of all human PDZ domains**. Clustering of related LNX PDZ domains is highlighted in a phylogenetic tree of all human PDZ domains. Click here for file

Additional file 4 **Conservation of ligand-binding residues in LNX1, LNX2 and MUPP1**. Table showing the amino acids identified as determinants of PDZ domain specificity for LNX1, LNX2 and MUPP1. Click here for file

Additional file 5 **Predicted interactions of LNX1, LNX2, MUPP1 & INADl PDZ domains**. Table showing predicted PDZ domain interactions for LNX1 PDZ1-4, LNX2 PDZ1-4, MUPP1 PDZ10-13 and INADl PDZ 8-10. Click here for file

Additional file 6 **Genbank accession numbers**. Table showing Genbank accession numbers for LNX and MUPP1 homologs referred to in this study. Click here for file
