# Supplementary material for: Molecular evolution of the LNX gene family
Source: BMC Evol Biol. 2011 Aug 9;11:235. doi: 10.1186/1471-2148-11-235 (PMC3162930; doi:10.1186/1471-2148-11-235)
Supplement: Additional file 1 — Analysis of splicing junctions in LNX1, LNX2, LNX3, LNX4 and MUPP1 PDZ domains. An alignment of PDZ domain sequences with splicing junctions highlighted. [file 1471-2148-11-235-S1.PDF]

Additional file 1

Analysis of splicing junctions in LNX1, LNX2, LNX3, LNX4, LNX5 and MUPP1 PDZ domains An alignment of human PDZ domain sequences from LNX1, LNX2, LNX3, LNX4 and MUPP1 prepared using ClustalW is shown with the positions of splicing junctions highlighted. Domains 10, 11 and 12 of MUPP1 have splicing junctions at exactly the same positions as domains 1, 2 and 3 respectively of LNX1 and LNX2. Splicing junctions in phylogenetically related domains are color coded as follows: LNX1/LNX2 PDZ1 and MUPP1 PDZ10 = Light Blue, LNX1/LNX2 PDZ2 and MUPP1 PDZ11 = Yellow, LNX1/LNX2 PDZ3 and MUPP1 PDZ12 = Pink, LNX1/LNX2 PDZ4 and MUPP1 PDZ13 Green, LNX3/LNX4/LNX5 = Dark Blue

```
MUPP1PDZ1       -----------EVFELLKPPSGGLGFSVVG-LRSENRGE------LGIFVQEIQEGSVAH 42
MUPP1PDZ2       -----------METIELVNDGSGLGFGIIG----GKAT--------GVIVKTILPGGVAD 37
MUPP1PDZ3       -----------TFDVELTKNVQGLGITIAGYIGDKKLEP------SGIFVKSITKSSAVE 43
MUPP1PDZ4       ----------VAHVSKFSE-NSGLGISLEA---TVGHH----------FIRSVLPEGPVG 36
MUPP1PDZ5       -----------IQHIELEKGSKGLGFSILDYQDPIDPAS------TVIIIRSLVPGGIAE 43
MUPP1PDZ6       -----------ERTINIAKGNSSLGMTVSA----NKDG-------LGMIVRSIIHGGAIS 38
MUPP1PDZ7       -----------RRVELWREPSKSLGISIVG-GRGMGSRLSNGEVMRGIFIKHVLEDSPAG 48
MUPP1PDZ8       -----------LHMIELEKGHSGLGLSLAG---NKDRSR------MSVFIVGIDPNGAAG 40
MUPP1PDZ9       -----------VQHLELPKDQGGLGIAIS---EEDTLS--------GVIIKSLTEHGVAA 38
MUPP1PDZ10      -----------ETTIEISKGRTGLGLSIVG---GSDTLL------GAIIIHEVYEEGAAC 40
MUPP1PDZ11      -----------LTIELQKKPGKGLGLSIVG-KRNDT----------GVFVSDIVKGGIAD 38
MUPP1PDZ12      -----------RTVEMKKGPTDSLGISIAG-GVGSPLGD------VPIFIAMMHPTGVAA 42
MUPP1PDZ13      IFQDDLGPPQCKSITLERGP-DGLGFSIVG-GYGSPHGD------LPIYVKTVFAKGAAS 52
LNX1PDZ1        -----------SIKINRVDPSESLSIRLVG---GSETPL------VHIIIQHIYRDGVIA 40
LNX1PDZ2        -----------HVILNKSSPEEQLGIKLVR--KVDEP---------GVFIFNVLDGGVAY 38
LNX1PDZ3        -----------KVVNIQKDPGESLGMTVAG-GASHREWD------LPIYVISVEPGGVIS 42
LNX1PDZ4        -----------KDIVLRRNTAGSLGFCIVG-GYEEYNGN------KPFFIKSIVEGTPAY 42
LNX2PDZ1        -----------TIEIHRSNPYIQLGISIVG---GNETPL------INIVIQEVYRDGVIA 40
LNX2PDZ2        -----------QVALHKRDSGEQLGIKLVR--RTDEP---------GVFILDLLEGGLAA 38
LNX2PDZ3        -----------KHITVKKEPHESLGMTVAG-GRGSKSGE------LPIFVTSVPPHGCLA 42
LNX2PDZ4        -----------HDIVLRRSYLGSWGFSIVG-GYEENHTN------QPFFIKTIVLGTPAY 42
LNX3PDZ1        -----------SLTLVLHRDSGSLGFNIIG-GRPSVDNH-DGSSSEGIFVSKIVDSGPAA 47
LNX3PDZ2        -----------EVDLYRMNSQDKLGLTVCY--RTDDEDD------IGIYISEIDPNSIAA 41
LNX4PDZ1        -----------PFTIVLERENDTLGFNIIG-GRPNQNNQ-EGTSTEGIYVSKILENGPAD 47
LNX4PDZ2        -----------EVELCRVSSQEKLGLTVCY--RTDDEED------TGIYVSEVDPNSIAA 41
LNX5PDZ         -----------EVELYKSSHRDKLGLMVCY--RTDDEED------LGIYVGEVNPNSIAA 41
                                        .: :                     :  :

MUPP1PDZ1       RDGRLKETDQILAINGQALDQTITHQQAISILQ---KAKDTVQ---LVIARGSLPQLVSP 96
MUPP1PDZ2       QHGRLCSGDHILKIGDTDLAG-MSSEQVAQVLR---QCGNRVK---LMIARGAIEERTAP 90
MUPP1PDZ3       HDGRIQIGDQIIAVDGTNLQG-FTNQQAVEVLR---HTGQTVL---LTLMRRGMKQEAEL 96
MUPP1PDZ4       HSGKLFSGDELLEVNGITLLG-ENHQDVVNILK---ELPIEVT---MVCCRRTVPPTTQS 89
MUPP1PDZ5       KDGRLLPGDRLMFVNDVNLEN-SSLEEAVEALKG--APSGTVR---IGVAKPLPLSPEEG 97
MUPP1PDZ6       RDGRIAIGDCILSINEESTIS-VTNAQARAMLRRHSLIGPDIK---ITYVPAEHLEEFKI 94
MUPP1PDZ7       KNGTLKPGDRIVEVDGMDLRD-ASHEQAVEAIR---KAGNPVV---FMVQSIINRPRKSP 101
MUPP1PDZ8       KDGRLQIADELLEINGQILYG-RSHQNASSIIK---CAPSKVK---IIFIRNKDAVNQMA 93
MUPP1PDZ9       TDGRLKVGDQILAVDDEIVVG-YPIEKFISLLK---TAKMTVK---LTIHAENPDSQAVP 91
MUPP1PDZ10      KDGRLWAGDQILEVNGIDLRK-ATHDEAINVLR---QTPQRVR---LTLYRDEAPYKEEE 93
MUPP1PDZ11      ADGRLMQGDQILMVNGEDVRN-ATQEAVAALLK---VSEGSLSSFTFPLSGSSTSESLES 94
MUPP1PDZ12      QTQKLRVGDRIVTICGTSTEG-MTHTQAVNLLK---NASGSIE---MQVVAGGDVSVVTG 95
MUPP1PDZ13      EDGRLKRGDQIIAVNGQSLEG-VTHEEAVAILK---RTKGTVT---LMVLS--------- 96
LNX1PDZ1        RDGRLLPGDIILKVNGMDISN-VPHNYAVRLLR---QPCQVLW---LTVMREQKFRSRNN 93
LNX1PDZ2        RHGQLEENDRVLAINGHDLRY-GSPESAAHLIQ---ASERRVH---LVVSRQVRQRSPDI 91
LNX1PDZ3        RDGRIKTGDILLNVDGVELTE-VSRSEAVALLK---RTSSS---IVLKALEVKEYEPQED 95
LNX1PDZ4        NDGRIRCGDILLAVNGRSTSG-MIHACLARLLK---ELKGRIT---LTIVSWPGTFL--- 92
LNX2PDZ1        RDGRLLAGDQILQVNNYNISN-VSHNYARAVLS---QPCNTLH---LTVLRERRFGNRAH 93
LNX2PDZ2        QDGRLSSNDRVLAINGHDLKY-GTPELAAQIIQ---ASGERVN---LTIARPGKPQPGNT 91
LNX2PDZ3        RDGRIKRGDVLLNINGIDLTN-LSHSEAVAMLK---ASAASPA-VALKALEVQIVEEATQ 97
LNX2PDZ4        YDGRLKCGDMIVAVNGLSTVG-MSHSALVPMLK---EQRNKVT---LTVICWPGSLV--- 92
LNX3PDZ1        KEGGLQIHDRIIEVNGRDLSR-ATHDQAVEAFK---TAKEPIV---VQVLRRTPRTKMFT 100
LNX3PDZ2        KDGRIREGDRIIQINGIEVQN--REEAVALLTS---EENKNFS---LLIARPELQLDEGW 93
LNX4PDZ1        RADGLEIHDKIMEVNGKDLSK-ATHEEAVEAFR---NAKEPIV---VQVLRRTPLSRPAY 100
LNX4PDZ2        KDGRIREGDRILQINGEDVQN--REEAVALLSN---DECKRIV---LLVARPEIQLDEGW 93
LNX5PDZ         KDGRIREGDRIIQINGVDVQN--REEAVAILSQ---EENTNIS---LLVARPESQLAKRW 93
                    :   * :: :                                .
```
