# Supplementary material for: Molecular evolution of the LNX gene family
Source: BMC Evol Biol. 2011 Aug 9;11:235. doi: 10.1186/1471-2148-11-235 (PMC3162930; doi:10.1186/1471-2148-11-235)
Supplement: Additional file 2 — Multiple sequence alignment of LNX3, LNX4 and LNX5 and their invertebrate orthologs. An alignment of full length murine LNX3, LNX4 and LNX5, as well as LNX3/4 orthologs from diverse invertebrate species. [file 1471-2148-11-235-S2.PDF]

Additional file 2

Multiple sequence alignment of LNX3, LNX4 and LNX5 and their invertebrate orthologs.

An alignment prepared using ClustalX of murine LNX3, LNX4 and LNX5, as well as LNX3/4 orthologs from diverse invertebrate species. Sequence conservation is plotted beneath the alignment and conserved residues marked and color coded according to the default CustalX settings. The alignment is also shown in FASTA format. Locations of protein domains within LNX3 are at the following positions in the alignment: RING = amino acid 18-56; PDZ1 = amino acid 260-342; PDZ2 = amino acid 463-540. Mm = *Mus musculus;* Sp = *Strongylocentrotus pupuratus;* Sk = *Saccoglossus kowalevskii;* Bf = *Branchiostoma floridae;* Am = *Apis mellifera;* Tc = *Tribolium castaneum ;* Ce = *Caenorhabditis elegans;* Dm = *Drosophila melanogaster;* Sm = *Schistosoma mansoni;* Hm = *Hydra magnipapillata*

```
Mm-LNX3      MGFELDRFDGDVDPDLKC-ALCHKVLEDPLTTPCGHVFCAGCVLPWVVQEGSCPARCRGRLSAKELNHVLPLKRLILKLD
Mm-LNX4      MGFALERLAEAVDPAFQCQLLCGQVLEEPACTPCGHVFCASCLLPWAAGRRWCPLQCQP-LAPGELYRVLPLRSLVQQLR
Mm-LNX5      --------------------------------------------------------------------------------
Sp-LNX34     MGFDIDRFSGPVDEDFKCSICLG-VLENPLATPCGHVFCSNCVLPWVVQNGSCPLKCEK-FSTKELNSVLPLRNLILKLE
Sk-LNX34     MGYDIERFCETIEENLKCSVCLG-VLEDPLATPCGHVFCSSCVLPWVVQNGNCPLKCEN-FSPKELNSVLPLRNIIQKLE
Bf-LNX34     --------------------------------------------------------------------------------
Tc-LNX34like --------------------------------------------------------------------------------
Am-LNX34like --------------------------------------------------------------------------------
Ce-LNX34like --------------------------------------------------------------------------------
Dm-Slip1     --------------------------------------------------------------------------------
Sm-LNX34like MLEFFKSWTTGFKKNTDQEPVNGQDLRNATHEQAIHAFQTAKEPIIVEVARRDPNVELLQKSAPNSNSGP----------
Hm-LNX34     MGIELERFVDSMVHDASCFICSCILEDPLEINPCGHHFCRGCWLNWSTKNSCCAFCGASVVGVKDSRLLWSLIQN-----
             1.......10........20........30........40........50........60........70........80

Mm-LNX3      IKCAHAARGCGRVVKLQDLPEHLERCDFAPARCRHAGCGQLLLRRDVEAHMRDACDARPVGRCQEGCGLPLTHGEQRAGG
Mm-LNX4      VQCNYSGQGCG----------------------HRGDCGCWQCTSSAAPSALPAAVTATAQAAAEAAGRKSTRRPRPAG-
Mm-LNX5      --------------------------------------------------------------------------------
Sp-LNX34     IRCDNFRRGCPEEVKIQMLAQHMEDCDFAPVKCSNKGCQDVINIKDQAQHETQTCEWRPVGRCEQGCSLVLQFN--TRSE
Sk-LNX34     VRCEYHRRGCNEMVKIHNLSQHVEDCDYLPIQCSNKGCRVVLNIKDQLQHETQTCEYRPVGQCKNGCGLTLLCR--DVHG
Bf-LNX34     --------------------------------------------------------------------------------
Tc-LNX34like --------------------------------------------------------------------------------
Am-LNX34like --------------------------------------------------------------------------------
Ce-LNX34like --------------------------------------------------------------------------------
Dm-Slip1     --------------------------------------------------------------------------------
Sm-LNX34like -------------------------VNSLDNQNKCSVAIQTDPSTAEATLAAMAAAALTTEDIRTALGIRHPGLPTSITF
Hm-LNX34     ----------------------------HNVYCIQKEKGCPAIYKFGFNKLHEGQCLYQGYNRVELNDKCTVCENDSGMT
             ........90.......100.......110.......120.......130.......140.......150.......160

Mm-LNX3      HCCARALRAHNGALQARLGALHKALKKEALRAGKREKSLVAQLAAAQLELQMTALRYQKKFTEYSARLDSLSRCVAAPPG
Mm-LNX4      --VGTRVRGHP-DLEVGL------WLSPAVGVGKTRRAQVSLLQR----------RYQEKLALYMA----HTRNFVGNPG
Mm-LNX5      --------------------------------------------------------------------------------
Sp-LNX34     HDCLKALQNHSGALQTKMKNQEHNLKKNSLRYGKREKALLAQIACLQNEIQMQALRYQKKLNESKAEMEYMSAVAS----
Sk-LNX34     HECLKALQAHVASQQVKLVSLEHDLKKMAIRYSKREKSLVVQLATLQNEIHMQALRYQKKLNEYKSQIQYLSKRATS---
Bf-LNX34     --------------------------------------------------------------------------------
Tc-LNX34like -----------------------------------------------------------MDSMLDCCYAGLDLYLSAPLC
Am-LNX34like --------------------------------------------------------------------------------
Ce-LNX34like --------------------------------------------------------------------------------
Dm-Slip1     ---------------------------------------------------------------MSIADVEYEYVVLKING
Sm-LNX34like KEENDSIIWDMGFHESENILIKEVTLCRKKPDEKFGLTLCYRQGDTSNTSCNVYVGELELNSLAEKSGQIYNGDQILSIN
Hm-LNX34     HDCLKELSLKMKQCSIRIINLEHENQHLSYKLMNREKEYLERISEIESQFYEESLKYNKEIR------DLRLRAAATQGE
             .......170.......180.......190.......200.......210.......220.......230.......240

Mm-LNX3      GKGE--ETKSLTLVLHRDSGSLGFNIIGGRPCVDNQDGSSSEGIFVSKIVDSGPAAKEGGLQIHDRIIEVNGKDLSRATH
Mm-LNX4      GRGQDGEQKPFTVVLEREDDTLGFNIIGGRPYQNSQKQSAPEGIYVSKILENGPADRADGLEVHDKIIAVNGRDLSKATH
Mm-LNX5      --------------------------MGCNMCVVQKPE-----------------------EQYKVMLQVNGKELSKLSQ
Sp-LNX34     FEKPWENTTILSLRLGRQDGSLGFNIIGGS-GISQGDGGISEGIIVSRVNEKGPADR-SQLQVHDRMIEVNGQDLSKATH
Sk-LNX34     AEKQPCEEATMSISLERENGSLGFNIMGGN-RVSIDGALPAEGIVVSRVTDHGPADR-GKLKVNDRIIQVNGKDLSRASH
Bf-LNX34     ----------------------------------------------------------------MACSVSQVNGKDLSKASH
Tc-LNX34like LHAANSDGSLQLWWLQPFSGLSARNALALHGRLCGLQHTRCLGASRSDAIDLCLQLN--ATYMDFIILKVNGTDVSSFPH
Am-LNX34like ---------------------------------------------------------------MDFVILKVNGQDVSNSSH
Ce-LNX34like -----------------------------------------------------------------------MASFTSPDI
Dm-Slip1     YDISHLSRYEAVQKFLQSKETLVVEIRRQKHNALDLELKHGSNAKISKVDNPGELSVLTDKSAEGTITAASASQQINCPS
Sm-LNX34like GNRIKSREHVIDLFQQSQLKVYKRPDQDSGMGRTTDESARTEESSEQEIDNDQHHHHHQYQQQQRTNFIHGLAQITKLTC
Hm-LNX34     QGKKNNQIYYVHIDLERLNGSLGFNIMGGA----TQDDKHSDGIIVSRIVEGGAADG--KLQLHDCILEVDSVDLRSATH
             .......250.......260.......270.......280.......290.......300.......310.......320
```

```
                 .           .:
Mm-LNX3        DQAVEAFKTAKEPIVVQVLRRTPRTK-MFTPAS----------ESQLVDTGTQTDITFEHIMALTKMSSPSPPVLD--PY
Mm-LNX4        EEAVEAFRTAKEPIVVQVLRRTPLSRPTYGMTP----------EVQLTNASTQTDITFEHIMALAKLRPLTPPVPDTCPF
Mm-LNX5        EQTLEALRASKEPLVIQVLRRSPRLR-GDSSCH----------DLQLVDSGTQTDITFEHIMALGKLRPPTPPMGILEPY
Sp-LNX34       EEAVEAFKNAQEPIIVQVMRRSACTAKNGSTTPNGQGSVPSPLERTVCSVGTQTELQNEDLLWKVLQRCSLPPSENHCDG
Sk-LNX34       EEAVEAFRNASEPIIVQVLRRTSRTKLK-------------SRESTTCSVSTQTDIQMDNFYTNSIHRCPTPPP---MSM
Bf-LNX34       EESVEAFRTAKEPIVVEVVRRSSVARQGTAKPD--------RREPCLVDAAVQTDIGPDPYYMLGMYRPPTPPPLSMQGT
Tc-LNX34like   EDAVRAFLTAQEPIVVEVKRRISESPQEKTPTS------------KLISTGCQTELSGLTWLEDNSLDCL----------
Am-LNX34like   EDAVRCFQSAQEPIIVEVLRRQPQQQQQQQQQQ------------QHHG---------------HRVHCA----------
Ce-LNX34like   DEKLAILHREMASLRVECDRLLNKHLIVEHTLS-----------------------------------------------
Dm-Slip1       STSLKEIET-KTPVVLTLRARSHEDRLGSLQAAS---------KETQTQSVVGTDVLKDNDLVNTITDNFIEHEHHLFEQ
Sm-LNX34like   ENGQFNNNSSGNSLTQDATSLDQKLAFPSDVFS----------DYDPIDPIDQELVQLGRLMQSLAVHCRQLVQAKMHYN
Hm-LNX34       ELAVKAFRESGNPVKMLVRRQISKSATSDFVDVG------------TQTFGVMFAVTCEEEFNEKHTNSFCKPQETLCKF
               .......330.......340.......350.......360.......370.......380.......390.......400
```

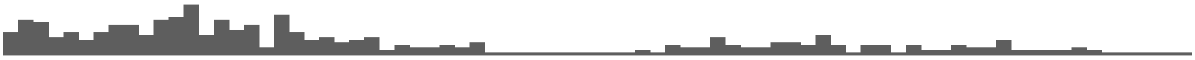

```
Mm-LNX3        LLP----EEHPASHDYYDPNDYMGDIHQD-------------MDR-EELELEEVGLYRMNSQDKLGLTVCYRTDD--EDD
Mm-LNX4        LLSDSCHSLHPMEHGFYEDHEYVSSLPAD-------------ADRADDFEYEEVELCRVSSQEKLGLTVCYRTDD--EED
Mm-LNX5        VLS----ELPPISHEYYDPAEFMEGGPQE-------------AERMDELEYEEVELCKNSHQDKLGLMVCYRTDE--EED
Sp-LNX34       TLDGLCDLDAEDPLSVLDSELLQKLEAMEGNLDLPILDEEDCLDRAYEMEYEDIVLKRSNTEEKLGLTLCYGS----EED
Sk-LNX34       SIKGLCEISPQQPMTLIDNDLLNELDSVD-NYDFPLLDEG--LDKGYEMEYEEVFLRRRNNNDKLGLTLCYGTDD--EEE
Bf-LNX34       APHP---YGDPMLYDAAEDLEFADDLPP--------------LDDNYNIEYEEVTLCRTTSEDKLGLTLCYGTDE--EED
Tc-LNX34like   ---------------------------------------------THDIDLEEVTLRKCSSDEKLGLTVCYSSGSEADTC
Am-LNX34like   ---------------------------------------------QEEKESERREHGTSNAWPNLVSTAVQTDWAGLIET
Ce-LNX34like   ------------------------------------------------------QSCSGWPNGAADTSSAYNTGG--ESC
Dm-Slip1       CLEPEIDIEEVTLVKG--------------------------VEQSSSNQIGLIVTSSGIQQSSTDTNKGDILGNVLEHS
Sm-LNX34like   RIQYPLPIIDNCTSQYGGNDSIVQPQTVLTKVITS------TTTSVINSSSIPTATTTTSSSSSLSSKISNQLTENCYYS
Hm-LNX34       SNQKRWLNPHEFT------------------------------ESINKEGVSMMSQQNSDTNKNEQYDSAYDTLLTQKSS
               .......410.......420.......430.......440.......450.......460.......470.......480
```

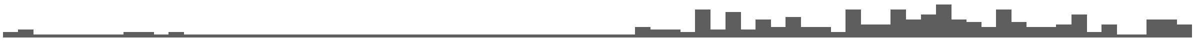

```
Mm-LNX3        IGIYISEIDPNSIAAKDGRIREGDRIIQINGIEVQNREEAVALLTSEENKNFSLLIARPELQLDEGWMDDDRNDFLDDLH
Mm-LNX4        TGIYVSEVDPNSIAAKDGRIREGDRILQINGEDIQNREEAVALLSSDECKRIVLLIARPDMQLDEGWLEDERNEFLEELN
Mm-LNX5        LGIYVGEVNPNSIAAKDGRIREGDRIIQINGMDVQNREEAVAILSQEENTNISLLVARPESQLAKRWKDSDRDDFLDDFG
Sp-LNX34       SGIFISEIDLNSIAYDDGRLKEGDQILQF-----------------------------------EDCLYEG-NALLDDLQ
Sk-LNX34       TGIFVSEIEPYSIAGQDGRIREGDQILQINGVDVHNKDQAIMLFSQDR-QEIAILVCRPQLQLDDGLLYDDTNVLLDDLH
Bf-LNX34       LGIYVSEINGEEVLNKDHAVR----------------------LFSEDKSEIKLLVARPDTRFDESWLEEERNMILDELN
Tc-LNX34like   TEVYIRDIAPQSVADRDGRLRQGDQILQVNGKD----------VANKEETESLFAENRKAVTLLVSRCYNRSEEYLDAEP
Am-LNX34like   EEEVVDEQPNDSFED----FLAHDIDFEVNGKD----------VANKEQTENLFAETKNAVTILVSRCLYQGSPLLSPDH
Ce-LNX34like   RSVSVTP---------------------------------------------------------------------DGVF
Dm-Slip1       EDVFISGVQPESIAYRDGRLRQGDQILRINGLD----------VKNQEELETQIARSSTSVTLLVSRILYPEDDDDEDIH
Sm-LNX34like   TGNNNLQNTKCIINKSNEGISNGSPLLSTKDNN------------HNMLTRLPRMGTRMEPSIMGSMSSNDSSGSFHDKN
Hm-LNX34       RSVRLDTTRNSQVSHDTTRVASPTDSIDKSTTR----------SSPVLKNNDNTSGVTLRNKIKTEQAENNRLSTFYILP
               .......490.......500.......510.......520.......530.......540.......550.......560
```

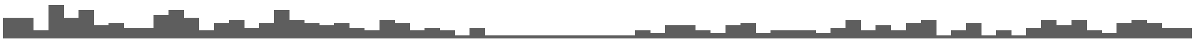

```
                                              .
Mm-LNX3        MDML-----EEQHHQAMQFTASVLQQ-KKHEEDGGTTDTATIL--SNQHEKDSGVGR-TDESTRNDESSEQENNGEDATA
Mm-LNX4        SELL-----EEDHNEGAQRTASELQQPKKQEEEEGTTDTATSS--SNNQEKDSGVGR-TDESLRNDESSEQENAVEEPHS
Mm-LNX5        SE-------NEGDLRARKLKSPPVQQIGNDEK--GAPDGGPGL--NNSQDLDSGVGR-TDESTRNEESSEHDLLGDEPPS
Sp-LNX34       MDML-----EQTHQEAMQFTACLLHSEGRPDDDG-TTDTGTTN-TSNHLEKDSGVGRATDESTRHDESSEQEILVDDASI
Sk-LNX34       MDML-----EQHHQEAMQFTSCLLDPECSHNDEGGTTDTGTTENTSTHHEKDSGVGR-TDESTRNDESSEQEILGDDCQK
Bf-LNX34       MHML-----EQHHSDAMRYTAAMLDYNECRHSKEEEGTTDSATSTSNQHEKDSGVGR-TDESTKNDESSEQDLGEDQ---
Tc-LNX34like   IDAI-----SPGGRSAAYQNSLIEQLVQQQTESQHSPRNDT-------APKVPPHFIQDRLNLTNSLVRSKMDSINHEIS
Am-LNX34like   IP--------------SYQNSLIEQLIRQQQQQTEERTKDSEEDGSTLRPPVPGHTPSSTCSSQASSQKGGGSGGRNGAS
Ce-LNX34like   IPTT-----SNSQMIQSQPSPRVLRRTVKMASPAVQRRLVPQNLPTSSSSRPPTVLLPHDSPEQPSRPIHR---------
Dm-Slip1       FEYANTFLPDDYTNVVDKLDKVLLTHVKSLEELSNKSAMQSDECYHIPEKNSSDSNVKISSLAKNIIEQSTKSCSKIKLR
Sm-LNX34like   LKMSAITTTGKLSHSRFPSNIKDRPVSSERHESQTQQQQQTTYCPDSNSKLIEPNSIYPDHLEQCRLNGSGDTSAYCTSE
Hm-LNX34       SDIS------AKEWNDVEKVVNFDYDFEYELEEGSEAETTGKVMVGDQIVQINGCQVKNVENAYKILQENETITLVVARA
               .......570.......580.......590.......600.......610.......620.......630.......640
```

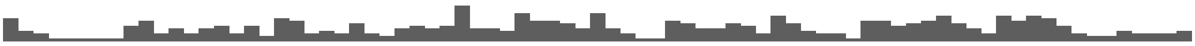

```
Mm-LNX3         SANPLAGQRKLTCSQDTLGSGDLPFSNESFISA--DCTDVDYLGIPEDECERFRELLELKCQVQSASPYSLYYP------
Mm-LNX4         TT--LKSKRELGKSQDTLGSLEHQCS-ESCAGG--ECLDSDCASNQE-VCEGFQQLLELK--IRNHGDYDLYYS------
Mm-LNX5         TTNTPGSLRKFGLQGDALQSRDFHFSMDSLLAEGAGLGGADLPGLTDEEYERYRELLEIKCHLENGNQLGIFFSR-----
Sp-LNX34        NQKFPVDTTSLG------SGGDLRYSNDSFTSNSTAEHDFMGHDISVEECMKFQAALESKCDNKHLRSDTDESMKGKETK
Sk-LNX34        NPGG--DSGSLG------SGGELRHSNDSFTSTDLPDQDFTGHEISVEECMKFKEALASKCDRSQCKSVPECNGRTNDT-
Bf-LNX34        ------SSHRLG-------SGELRNSNSSFTSN----EQEFGQEITAEQCAQFQKELERKCNESYGTRDTGTLSDVS---
Tc-LNX34like    ELDHRMQNIQLVK------IDKRKPPQLPHIPAPQES---DTEHIYETIPESVDSEMEPIYSCPYEADEQSMVEQWL---
Am-LNX34like    PARHACD-HERGK------QQQWVQEAMKDCSKKIESLCVETEHIYETIPE---SDSEPIYSSPYEHHHR----------
Ce-LNX34like    --------------------------------------------------------------------------------
Dm-Slip1        PNANLDYKKKFNLPLSQEEVHLQYEYDESEHIYETIPEDSESEPVYCSPYQRSNDKTSIGCSSPIASRPAESLER-----
Sm-LNX34like    REGITASLSDLGGSLLSLSAVIPDPHHYHHHRHHSRPQYNSSEVNSTNHQITPNDSFSPSSNCPSNEHKRNMNSTKDLS-
Hm-LNX34        VYEQQSDCFDEAEETDLDLLDSINEEAESHYLSLSNPVKLLDNATYDNKAFSYKNHISEKCLHANEKNLGIAPVTN----
                .......650.......660.......670.......680.......690.......700.......710.......720
```

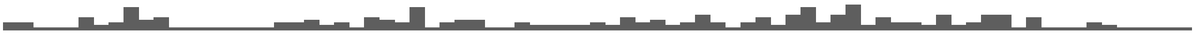

```
Mm-LNX3         --------------------SSPLDAAGKSDPESVDKELELLNEELRS--------IELECLSIVRAHKMQQLKEQYRES
Mm-LNX4         --------------------SSTIECN-RGEQDGVEHELQLLNEELRN--------IELECQNIMQAHRLQKVTDQYGDI
Mm-LNX5         -------------------ASSGNSALDVNRNESLGHEMAMLEEELRH--------LEFKCRNILRAQKMQQLRERCMKA
Sp-LNX34        VMRLGSDSMDTTSQSGDSLNRPCRNRLICDQQDELQKERRINGHDVWLSNHAQDEQLDTIPCQCPSSESSQKVDMPEKDN
Sk-LNX34        ---IG----ATDSSSRDYIHHLHNSDLPLYADDNLGYNNVVYEGDISR----QTDDDVWVAQDFARSHEIILTTIPEDQL
Bf-LNX34        --------------------ERELEVLNRRMEGMSFHTMPHYPRDVTP-----YDNLYENIMYYHQRNTPDVLSSYENLG
Tc-LNX34like    --------------------KLQDQGWNPQNKDATPKDNKKQRCKSSK--------SNSSGEEHENSSSAYNTGGSCNSN
Am-LNX34like    ------------------------HHWHHSS------NNHHGPSVVAT--------AQTTTVSTNASANAINVASSTVIQ
Ce-LNX34like    ----------------------------------MHQELPIPEDVVS--------------MVSSNKTQRSIIRAPSAVF
Dm-Slip1        --------------------TMQQQTQRVAQWLGLKPQYQKTRQTLVG---------RPPPLKLVQQPTCSRVFTLRSTL
Sm-LNX34like    ------------------IRSDFRHQHHQHHHPKLHNSNLITEHNPQK--------FVKEDKSSSPSSIHPNKTKNLQKY
Hm-LNX34        --------------------NNNDNNIERLREEIIDNTKSEKMTSVLKN----------FVRNKFGNSEKTDKRSSCGSD
                .......730.......740.......750.......760.......770.......780.......790.......800
```

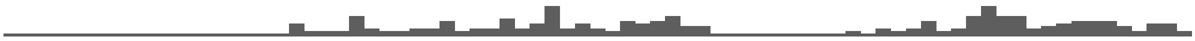

```
Mm-LNX3         WMLHHSGFRNYNTSVDVRRHELSDITELPEKSDKDSSSAYNTGESCRSTPLTLEISPDNSLRR---------VAEGSSE-
Mm-LNX4         WALHDGGFRNYNTSLDVQRGKLDDIMEHPEKSDKDSSSAYNTAESCRSTPLTVDRSPDSSLPR---------MINLTNKK
Mm-LNX5         WLLEEESLYDLAAS-EPKKHELSDISELPEKSDKDSTSAYNTGESCRSTPLLVEPLPESPLKR---------SGAGNSNL
Sp-LNX34        SHQRQEDLSPETKEEANKENGLPSNERQHRRSSSGSGRSHRAPRTVPGSPGGRSKSSSSSRDR---------SKSTSSSG
Sk-LNX34        SLKR---MSSDSEKEKQGKLGREPSQESIQTEELGQARAALTLHLRSTEFEQTRNKDRNRDS----------SSEKESSG
Bf-LNX34        HGLRHPGPHSVGPKPDIVQPEMTEIEKEDKQSD-NSSSAYNTGESCRSTPLTLEFSVDSCKRSDTSSENVVTVVSTQPTA
Tc-LNX34like    PLTFELACSQDSKQKDTYRSTLILCPPVEKEDTSKEKDQTETCDACKQQATSKKKSKSTSKTN-------------SPSS
Am-LNX34like    YTQTGQTVSQTSAGR-WYCSSKSNSSGEEKDSSSAYN----TGESNNSSSNNNNSSRDSGRVS-------------SSQY
Ce-LNX34like    GALKGSSLRKLSISRNIQMTTKVNKEESDRKEKEAEEYAKRFRIPSYSVVLKEEKRKSSFRIP-----------------
Dm-Slip1        TNTSASSSSGVAYSSYGQNNVVTGNAAAPGEEVDNSSSAYNTGDSNNSASPHQNTTNPDEAIATG--RKLDSTVIDSPND
Sm-LNX34like    SQLHNHPLYPLPPLYCNECYYPIKDISPMKCNTSNINEQLSIINTSIPSIINKNLHSYEFYDNFYCSLTNKTIMTTNNEK
Hm-LNX34        ESKDQNKIPRCDSNSSSTSGKSIKTQLNEESAFIKEQKHLSKMKAYLDSPSIARHRVNQKIIQ--------------PRS
                .......810.......820.......830.......840.......850.......860.......870.......880
```

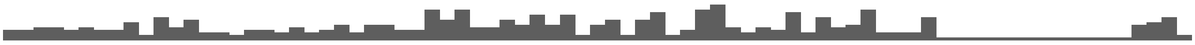

```
Mm-LNX3         GATANIEAYRPSPKNLLAITEDPEVSTPSYNPSAKELDPSQALEIKERRGS--------------------DGSRSPTAS
Mm-LNX4         NLRSMMTAHQSPPRQSTREYTSTKVKATDEGCSVESLEKG--LESSQLLDQ--------------------EHTVSEHPP
Mm-LNX5         NRTPSGPPVTTHLKGAPSPGSPAKFRSLSRDPEVGRRQHT---EERVRRST--------------------KTSVTLERV
Sp-LNX34        STKGSGKDKHSSPSKGASTNNGETNHKHQQTQQQQQQQQQQQQQDHLGQLPGAKAYPRSPATNPHLRAQFGQRSPYLMQH
Sk-LNX34        SGKQINKCGTPTKHDGKSQRAGSPSKSKPKEKSGAKERMSSPSKDKRDSNQ--NQISSSHHQKNSNRKQVSNDNGGINQQ
Bf-LNX34        SVKVTETSNDSRPSQSPVRKLGKPDSVKDLRSRSTSREKEGKVEKKHKDTKETQKSLRREKPPRRDRQQEQSVYFQPRPR
Tc-LNX34like    PTTRIGVGATPAHLLSDTMYTNVANLQQTMLLQQQLFRQALVQNNIDIASK-----------------------------
Am-LNX34like    YKDRVTDATGAGNLPADTMYTNIANLQQTMLLQQQLFKQALDRRNSTIAGS-----------------------------
Ce-LNX34like    ----VPHYFRSSKAKSKVYQVDAPDEEIEEVIDEEKMAAKTDLGDTTVHYK-----------------------------
Dm-Slip1        HLDATGVSTMLLLPFGKSGRIGLCSSNLPTAYVSERYTNVGSENEIHPLKS-----------------------------
Sm-LNX34like    EQSILNQSFKQFNKLINIDKTNIINEEPTLSDIYETPYASVTIHDEPIGDQYP-----------------------MKCI
Hm-LNX34        SINLETYQAISCRNTKEVDQCNLPVKQFSSTYTLPCTENTDKSLNNRRYCDS----------------------------
                .......890.......900.......910.......920.......930.......940.......950.......960
```

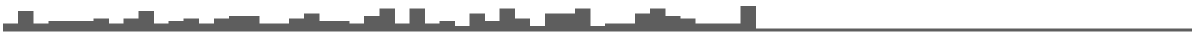

```
Mm-LNX3      PKLGNAYLPSYHHSPYKHAHIPAHAQHYQSYMHLIQQKSAVEYAQSQMSLVS---------------------MCKDLNS
Mm-LNX4      ------YLSPYHSSSYRYANIPAHARHYQSYMQLIQQKSAVEYAQSQLSLVS---------------------MCKESQR
Mm-LNX5      GPEGSPYLSRRHRG--------QEIEQYHSCVQLAPPRTLEDLGHGSLSLASG-------------------PRVGGVAA
Sp-LNX34     DSTRTNLLKHNWKVQQQASQLKRSFDKYGSIQSLQSVPGYAKHYRSYLHLINQNE-----------------ENAMKSQN
Sk-LNX34     NS---CLREQNRKLHEQNMHLRRSMHRLSEVG--MSVPSYAQHYQSYLHLIQQRE-----------------DMSLSSAV
Bf-LNX34     FVERDSVYFQPRNRPSMYASQQSIRTMSSMCTSQMSIPVHAQHYRSYMHLLKAQEREMCRKARQKEVEKEPPREQVREEE
Tc-LNX34like ---------PTTS---------------------FTSPSLSQYQFVSSSQTYTT-----------------------QPE
Am-LNX34like ---------RESSSVTAVAAVPTSAAKKSRSHGNFQAPNLTRYQFVGSQQVCASS------------------NSWIPPE
Ce-LNX34like --------------------------------------------------------------------------------
Dm-Slip1     ----DIEILRVKPTDDSYSHCPQFNAPNLSSYHFVSSQEVANRCHISTSLQKN---------------------ATLLNG
Sm-LNX34like SKDPCYPLVYPHPMYTSATCHSKRYQNTAVSASVITGAVSSSSSTAATSTITTTG-----------------MPPSGQVY
Hm-LNX34     ------ISSLRSSMKTNLVSNKTKPEKHVQIDSHPGWNATEKRVWEEFKIIQS--------------------MEAKENS
             .......970.......980.......990......1000......1010......1020......1030......1040
```

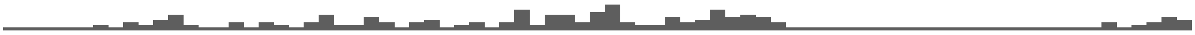

```
                 * *:    **.:   ::.                          .:          .:  :     :*:*::.:  :   :*   :.::
Mm-LNX3      SNSVEPRMEWKVKIRSDGTRYITKRPV-------------RDRLLRERALKIREERSGLTTDDDAMSEMKMGRYWSKEER
Mm-LNX4      GS--EPKMEWKVKIRSDGTRYITKRPV-------------RDRLLKERALKIKEERSGMTTDDDTMSEMKMGRYWSKEER
Mm-LNX5      AAVEAPRMEWKVKVRSDGTRYVAKRPV-------------RDRLLKARALKIREERSGMTTDDDAVSEMKMGRYWSKEER
Sp-LNX34     NSQGNSEGQWKVKIRSDGTRYITKRSS-------------RDRLLKERATKIQEERAGMTTDDEAVSEMKLGRYWSKEDR
Sk-LNX34     KKE-KPDTEWKVKVRSDGTRYITRRPV-------------RDRLLKQRANRIREERSGMTTDDDAVSEVKTGRYWSKEDK
Bf-LNX34     KPEDDGNFEWKVKIRSDGSRYITRRPV-------------RDRLLKERAMKIQEERMGLTTDDEAISEMKLGRYWTREER
Tc-LNX34like TATLETMMEWKVKRRPDGTRYIARRPV-------------RNRILKNRAIKISEERAGLTTEDDTVSELKIGRYWTKEER
Am-LNX34like EKGNEVQMEWKVKRRADGTRYIARRPV-------------RNRILRNRAIKISEERAGHSTEDDTMSEVKIGRYWTKEER
Ce-LNX34like ---------WKVKRRCDGSRYIVKRPI-------------RSQILKKREAQVYRERAPISTDDDAMSELKLGRYHTKEER
Dm-Slip1     ESAEEIPMVWKVKRRPDGTRYIVKRPV-------------RNRPQVALRKNMRYN-EVTTTEDDTISEVKIGRYWTKEER
Sm-LNX34like PMDNWNMMEWVVKKRPDGTRYITRRPISTSTVTTIFADNDSNQLVEERAKRLAEERSGITTDDDAISELKTGRYWNRSER
Hm-LNX34     LDPTKIEAEWRVRRSKDGKHVYIKKTNS-----------NRNKVLKEREDEINKERCGMTTDDDAFTIYQG-QYWDRDQR
             ......1050......1060......1070......1080......1090......1100......1110......1120
```

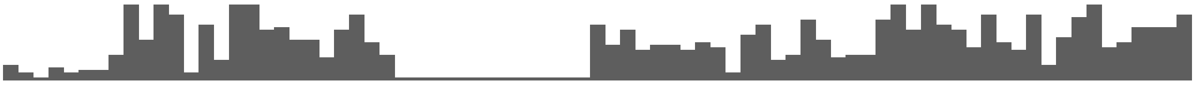

```
             :. : : .  :
Mm-LNX3      KQHLVKAKEQRRRREFMMQSRLDCLKE--QQA--SDDRKEMNILELS---------HKKMMKK-------------RNKK-
Mm-LNX4      KQHLVRAKEQRRRREFMMRSRLESLKESPQSS--GEGKKELSIIELS---------HKKMMKK-------------RNKK-
Mm-LNX5      KQHLIRAREQRKRREFMMQSRLECLRE--QQN--GDSKPELNIIALS---------HRKTMKK-------------RNKK-
Sp-LNX34     KRHLERAKDQRLRREYMQQARMECLQELEEDILSGGGKREPDIVELS---------HRKLNKR-------------KGRKM
Sk-LNX34     KRHLERARDQKQRKEFMIRARMESLKEQNED---GDAIKKESIVELS---------HKKMLKK-------------KGRK-
Bf-LNX34     REHLQRAKDQKRRREFMERARLDILKE---------SNNENPILEMS---------QKKMSKK-------------GKGK-
Tc-LNX34like KKHLEKSKERKQRQE--ILLASRNIEENGELVIHKPVEKVHSFSSK-----KAANNLDNTVKK--------HKSKRAHKE
Am-LNX34like KRQLERARERKQRQQEFQLLQQQQQQQQQQQQQQQQQQQQQQLQQTNELLASEHGEHEKTCKKPLNILELSHKKMARKKN
Ce-LNX34like KRILERENTKKMLKLQQKMMEKAHPSE-------------QVIYQMS---------QQKLARQ-------------KDAM
Dm-Slip1     KRHIERAREKRHHQTQQQQQ------------------------------------------------------------
Sm-LNX34like KKQLEKAKADRKRKQLNIITQQNSHKN-----------------------------------------------------
Hm-LNX34     KRQLVRHQDRRQKLLEKAAIKATYQNET--------GRKIAEFVQRN---------------------------MTLPGA
             ......1130......1140......1150......1160......1170......1180......1190......1200
```

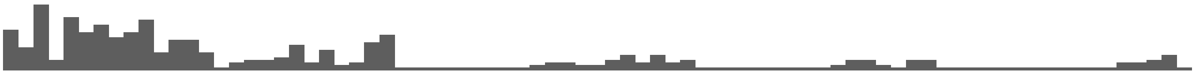

```
Mm-LNX3      IFDNWMTIQELLTHGTKS-------PDGTRVYNSFLSVTTV
Mm-LNX4      ILDNWMTIQELMTHGAKS-------PDGTRVHNAFLSVTTV
Mm-LNX5      ILDNWITIQEMLAHGARS-------ADGKRIYNPLLSVTTV
Sp-LNX34     MLDDFTTVQEMLIHGSKVS------PDSAKEFSPFLNVTMV
Sk-LNX34     IFDDFVTVQEMLAHGSKLS------PETLKQFNPLLNVTTV
Bf-LNX34     IMDNFMTVQELLAQRVTD---------GSKTLGPLVTLTTV
Tc-LNX34like GYDNFPTVQEMIVHGPKVP--------TNGKMMGLLSVTTV
Am-LNX34like TLDDFTTVQEMLVHGNRVGGTGGPGGGTGGKLMGLLSVTTV
Ce-LNX34like VMDEFVTTREVLSSRTRPD-----------GIHGVVSVTTV
Dm-Slip1     -----------------------------------------
Sm-LNX34like --LVLTTTNSSPINKSDGN------------NTTLVTMTTV
Hm-LNX34     VFDNFITIEEILSQRNRSG-----------IFNGPIHVTTI
             ......1210......1220......1230......1240.
```

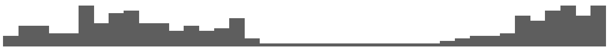

Fig S1. Alignment files in FASTA format.

```
>Mm-LNX3
MGFELDRFDGDVDPDLKC-ALCHKVLEDPLTTPCGHVFCAGCVLPWVVQEGSCPARCRGR
LSAKELNHVLPLKRLILKLDIKCAHAARGCGRVVKLQDLPEHLERCDFAPARCRHAGCGQ
LLLRRDVEAHMRDACDARPVGRCQEGCGLPLTHGEQRAGGHCCARALRAHNGALQARLGA
LHKALKKEALRAGKREKSLVAQLAAAQLELQMTALRYQKKFTEYSARLDSLSRCVAAPPG
GKGE--ETKSLTLVLHRDSGSLGFNIIGGRPCVDNQDGSSSEGIFVSKIVDSGPAAKEGG
LQIHDRIIEVNGKDLSRATHDQAVEAFKTAKEPIVVQVLRRTPRTK-MFTPAS-------
---ESQLVDTGTQTDITFEHIMALTKMSSPSPPVLD--PYLLP----EEHPASHDYYDPN
DYMGDIHQD-------------MDR-EELELEEVGLYRMNSQDKLGLTVCYRTDD--EDD
IGIYISEIDPNSIAAKDGRIREGDRIIQINGIEVQNREEAVALLTSEENKNFSLLIARPE
LQLDEGWMDDDRNDFLDDLHMDML-----EEQHHQAMQFTASVLQQ-KKHEEDGGTTDTA
TIL--SNQHEKDSGVGR-TDESTRNDESSEQENNGEDATASANPLAGQRKLTCSQDTLGS
GDLPFSNESFISA--DCTDVDYLGIPEDECERFRELLELKCQVQSASPYSLYYP------
--------------------SSPLDAAGKSDPESVDKELELLNEELRS--------IELE
CLSIVRAHKMQQLKEQYRESWMLHHSGFRNYNTSVDVRRHELSDITELPEKSDKDSSSAY
NTGESCRSTPLTLEISPDNSLRR---------VAEGSSE-GATANIEAYRPSPKNLLAIT
EDPEVSTPSYNPSAKELDPSQALEIKERRGS--------------------DGSRSPTAS
PKLGNAYLPSYHHSPYKHAHIPAHAQHYQSYMHLIQQKSAVEYAQSQMSLVS--------
-------------MCKDLNSSNSVEPRMEWKVKIRSDGTRYITKRPV-------------
RDRLLRERALKIREERSGLTTDDDAMSEMKMGRYWSKEERKQHLVKAKEQRRRREFMMQS
RLDCLKE--QQA--SDDRKEMNILELS---------HKKMMKK------------RNKK-
IFDNWMTIQELLTHGTKS-------PDGTRVYNSFLSVTTV
>Mm-LNX4
MGFALERLAEAVDPAFQCQLLCGQVLEEPACTPCGHVFCASCLLPWAAGRRWCPLQCQP-
LAPGELYRVLPLRSLVQQLRVQCNYSGQGCG----------------------HRGDCGC
WQCTSSAAPSALPAAVTATAQAAAEAAGRKSTRRPRPAG---VGTRVRGHP-DLEVGL--
----WLSPAVGVGKTRRAQVSLLQR----------RYQEKLALYMA----HTRNFVGNPG
GRGQDGEQKPFTVVLEREDDTLGFNIIGGRPYQNSQKQSAPEGIYVSKILENGPADRADG
LEVHDKIIAVNGRDLSKATHEEAVEAFRTAKEPIVVQVLRRTPLSRPTYGMTP-------
---EVQLTNASTQTDITFEHIMALAKLRPLTPPVPDTCPFLLSDSCHSLHPMEHGFYEDH
EYVSSLPAD-------------ADRADDFEYEEVELCRVSSQEKLGLTVCYRTDD--EED
TGIYVSEVDPNSIAAKDGRIREGDRILQINGEDIQNREEAVALLSSDECKRIVLLIARPD
MQLDEGWLEDERNEFLEELNSELL-----EEDHNEGAQRTASELQQPKKQEEEEGTTDTA
TSS--SNNQEKDSGVGR-TDESLRNDESSEQENAVEEPHSTT--LKSKRELGKSQDTLGS
LEHQCS-ESCAGG--ECLDSDCASNQE-VCEGFQQLLELK--IRNHGDYDLYYS------
--------------------SSTIECN-RGEQDGVEHELQLLNEELRN--------IELE
CQNIMQAHRLQKVTDQYGDIWALHDGGFRNYNTSLDVQRGKLDDIMEHPEKSDKDSSSAY
NTAESCRSTPLTVDRSPDSSLPR---------MINLTNKKNLRSMMTAHQSPPRQSTREY
TSTKVKATDEGCSVESLEKG--LESSQLLDQ--------------------EHTVSEHPP
------YLSPYHSSSYRYANIPAHARHYQSYMQLIQQKSAVEYAQSQLSLVS--------
-------------MCKESQRGS--EPKMEWKVKIRSDGTRYITKRPV-------------
RDRLLKERALKIKEERSGMTTDDDTMSEMKMGRYWSKEERKQHLVRAKEQRRRREFMMRS
RLESLKESPQSS--GEGKKELSIIELS---------HKKMMKK------------RNKK-
ILDNWMTIQELMTHGAKS-------PDGTRVHNAFLSVTTV
>Mm-LNX5
------------------------------------------------------------
------------------------------------------------------------
------------------------------------------------------------
------------------------------------------------------------
--------------------------MGCNMCVVQKPE----------------------
-EQYKVMLQVNGKELSKLSQEQTLEALRASKEPLVIQVLRRSPRLR-GDSSCH-------
---DLQLVDSGTQTDITFEHIMALGKLRPPTPPMGILEPYVLS----ELPPISHEYYDPA
EFMEGGPQE-------------AERMDELEYEEVELCKNSHQDKLGLMVCYRTDE--EED
LGIYVGEVNPNSIAAKDGRIREGDRIIQINGMDVQNREEAVAILSQEENTNISLLVARPE
SQLAKRWKDSDRDDFLDDFGSE-------NEGDLRARKLKSPPVQQIGNDEK--GAPDGG
PGL--NNSQDLDSGVGR-TDESTRNEESSEHDLLGDEPPSTTNTPGSLRKFGLQGDALQS
RDFHFSMDSLLAEGAGLGGADLPGLTDEEYERYRELLEIKCHLENGNQLGIFFSR-----
-------------------ASSGNSALDVNRNESLGHEMAMLEEELRH--------LEFK
```

```
CRNILRAQKMQQLRERCMKAWLLEEESLYDLAAS-EPKKHELSDISELPEKSDKDSTSAY
NTGESCRSTPLLVEPLPESPLKR---------SGAGNSNLNRTPSGPPVTTHLKGAPSPG
SPAKFRSLSRDPEVGRRQHT---EERVRRST--------------------KTSVTLERV
GPEGSPYLSRRHRG--------QEIEQYHSCVQLAPPRTLEDLGHGSLSLASG-------
------------PRVGGVAAAAVEAPRMEWKVKVRSDGTRYVAKRPV-------------
RDRLLKARALKIREERSGMTTDDDAVSEMKMGRYWSKEERKQHLIRAREQRKRREFMMQS
RLECLRE--QQN--GDSKPELNIIALS---------HRKTMKK------------RNKK-
ILDNWITIQEMLAHGARS-------ADGKRIYNPLLSVTTV
>Sp-LNX34
MGFDIDRFSGPVDEDFKCSICLG-VLENPLATPCGHVFCSNCVLPWVVQNGSCPLKCEK-
FSTKELNSVLPLRNLILKLEIRCDNFRRGCPEEVKIQMLAQHMEDCDFAPVKCSNKGCQD
VINIKDQAQHETQTCEWRPVGRCEQGCSLVLQFN--TRSEHDCLKALQNHSGALQTKMKN
QEHNLKKNSLRYGKREKALLAQIACLQNEIQMQALRYQKKLNESKAEMEYMSAVAS----
FEKPWENTTILSLRLGRQDGSLGFNIIGGS-GISQGDGGISEGIIVSRVNEKGPADR-SQ
LQVHDRMIEVNGQDLSKATHEEAVEAFKNAQEPIIVQVMRRSACTAKNGSTTPNGQGSVP
SPLERTVCSVGTQTELQNEDLLWKVLQRCSLPPSENHCDGTLDGLCDLDAEDPLSVLDSE
LLQKLEAMEGNLDLPILDEEDCLDRAYEMEYEDIVLKRSNTEEKLGLTLCYGS----EED
SGIFISEIDLNSIAYDDGRLKEGDQILQF-------------------------------
----EDCLYEG-NALLDDLQMDML-----EQTHQEAMQFTACLLHSEGRPDDDG-TTDTG
TTN-TSNHLEKDSGVGRATDESTRHDESSEQEILVDDASINQKFPVDTTSLG------SG
GDLRYSNDSFTSNSTAEHDFMGHDISVEECMKFQAALESKCDNKHLRSDTDESMKGKETK
VMRLGSDSMDTTSQSGDSLNRPCRNRLICDQQDELQKERRINGHDVWLSNHAQDEQLDTI
PCQCPSSESSQKVDMPEKDNSHQRQEDLSPETKEEANKENGLPSNERQHRRSSSGSGRSH
RAPRTVPGSPGGRSKSSSSSRDR---------SKSTSSSGSTKGSGKDKHSSPSKGASTN
NGETNHKHQQTQQQQQQQQQQQQQDHLGQLPGAKAYPRSPATNPHLRAQFGQRSPYLMQH
DSTRTNLLKHNWKVQQQASQLKRSFDKYGSIQSLQSVPGYAKHYRSYLHLINQNE-----
------------ENAMKSQNNSQGNSEGQWKVKIRSDGTRYITKRSS-------------
RDRLLKERATKIQEERAGMTTDDEAVSEMKLGRYWSKEDRKRHLERAKDQRLRREYMQQA
RMECLQELEEDILSGGGKREPDIVELS---------HRKLNKR------------KGRKM
MLDDFTTVQEMLIHGSKVS------PDSAKEFSPFLNVTMV
>Sk-LNX34
MGYDIERFCETIEENLKCSVCLG-VLEDPLATPCGHVFCSSCVLPWVVQNGNCPLKCEN-
FSPKELNSVLPLRNIIQKLEVRCEYHRRGCNEMVKIHNLSQHVEDCDYLPIQCSNKGCRV
VLNIKDQLQHETQTCEYRPVGQCKNGCGLTLLCR--DVHGHECLKALQAHVASQQVKLVS
LEHDLKKMAIRYSKREKSLVVQLATLQNEIHMQALRYQKKLNEYKSQIQYLSKRATS---
AEKQPCEEATMSISLERENGSLGFNIMGGN-RVSIDGALPAEGIVVSRVTDHGPADR-GK
LKVNDRIIQVNGKDLSRASHEEAVEAFRNASEPIIVQVLRRTSRTKLK------------
-SRESTTCSVSTQTDIQMDNFYTNSIHRCPTPPP---MSMSIKGLCEISPQQPMTLIDND
LLNELDSVD-NYDFPLLDEG--LDKGYEMEYEEVFLRRRNNNDKLGLTLCYGTDD--EEE
TGIFVSEIEPYSIAGQDGRIREGDQILQINGVDVHNKDQAIMLFSQDR-QEIAILVCRPQ
LQLDDGLLYDDTNVLLDDLHMDML-----EQHHQEAMQFTSCLLDPECSHNDEGGTTDTG
TTENTSTHHEKDSGVGR-TDESTRNDESSEQEILGDDCQKNPGG--DSGSLG------SG
GELRHSNDSFTSTDLPDQDFTGHEISVEECMKFKEALASKCDRSQCKSVPECNGRTNDT-
---IG----ATDSSSRDYIHHLHNSDLPLYADDNLGYNNVVYEGDISR----QTDDDVWV
AQDFARSHEIILTTIPEDQLSLKR---MSSDSEKEKQGKLGREPSQESIQTEELGQARAA
LTLHLRSSTEFEQTRNKDRNRDS---------SSEKESSGSGKQINKCGTPTKHDGKSQR
AGSPSKSKPKEKSGAKERMSSPSKDKRDSNQ--NQISSSHHQKNSNRKQVSNDNGGINQQ
NS---CLREQNRKLHEQNMHLRRSMHRLSEVG--MSVPSYAQHYQSYLHLIQQRE-----
------------DMSLSSAVKKE-KPDTEWKVKVRSDGTRYITRRPV-------------
RDRLLKQRANRIREERSGMTTDDDAVSEVKTGRYWSKEDKKRHLERARDQKQRKEFMIRA
RMESLKEQNED---GDAIKKESIVELS---------HKKMLKK------------KGRK-
IFDDFVTVQEMLAHGSKLS------PETLKQFNPLLNVTTV
>Bf-LNX34
------------------------------------------------------------
------------------------------------------------------------
------------------------------------------------------------
------------------------------------------------------------
------------------------------------------------------------
--MACSVSQVNGKDLSKASHEESVEAFRTAKEPIVVEVVRRSSVARQGTAKPD-------
-RREPCLVDAAVQTDIGPDPYYMLGMYRPPTPPPLSMQGTAPHP---YGDPMLYDAAEDL
```

```
EFADDLPP--------------LDDNYNIEYEEVTLCRTTSEDKLGLTLCYGTDE--EED
LGIYVSEINGEEVLNKDHAVR----------------------LFSEDKSEIKLLVARPD
TRFDESWLEEERNMILDELNMHML-----EQHHSDAMRYTAAMLDYNECRHSKEEEGTTD
SATSTSNQHEKDSGVGR-TDESTKNDESSEQDLGEDQ--------SSHRLG--------S
GELRNSNSSFTSN----EQEFGQEITAEQCAQFQKELERKCNESYGTRDTGTLSDVS---
--------------------ERELEVLNRRMEGMSFHTMPHYPRDVTP-----YDNLYEN
IMYYHQRNTPDVLSSYENLGHGLRHPGPHSVGPKPDIVQPEMTEIEKEDKQSD-NSSSAY
NTGESCRSTPLTLEFSVDSCKRSDTSSENVVTVVSTQPTASVKVTETSNDSRPSQSPVRK
LGKPDSVKDLRSRSTSREKEGKVEKKHKDTKETQKSLRREKPPRRDRQQEQSVYFQPRPR
FVERDSVYFQPRNRPSMYASQQSIRTMSSMCTSQMSIPVHAQHYRSYMHLLKAQEREMCR
KARQKEVEKEPPREQVREEEKPEDDGNFEWKVKIRSDGSRYITRRPV-------------
RDRLLKERAMKIQEERMGLTTDDEAISEMKLGRYWTREERREHLQRAKDQKRRREFMERA
RLDILKE---------SNNENPILEMS---------QKKMSKK------------GKGK-
IMDNFMTVQELLAQRVTD---------GSKTLGPLVTLTTV
>Tc-LNX34like
------------------------------------------------------------
------------------------------------------------------------
------------------------------------------------------------
---------------------------------------MDSMLDCCYAGLDLYLSAPLC
LHAANSDGSLQLWWLQPFSGLSARNALALHGRLCGLQHTRCLGASRSDAIDLCLQLN--A
TYMDFIILKVNGTDVSSFPHEDAVRAFLTAQEPIVVEVKRRISESPQEKTPTS-------
-----KLISTGCQTELSGLTWLEDNSLDCL------------------------------
-------------------------THDIDLEEVTLRKCSSDEKLGLTVCYSSGSEADTC
TEVYIRDIAPQSVADRDGRLRQGDQILQVNGKD----------VANKEETESLFAENRKA
VTLLVSRCYNRSEEYLDAEPIDAI-----SPGGRSAAYQNSLIEQLVQQQTESQHSPRND
T-------APKVPPHFIQDRLNLTNSLVRSKMDSINHEISELDHRMQNIQLVK------I
DKRKPPQLPHIPAPQES---DTEHIYETIPESVDSEMEPIYSCPYEADEQSMVEQWL---
--------------------KLQDQGWNPQNKDATPKDNKKQRCKSSK--------SNSS
GEEHENSSSAYNTGGSCNSNPLTFELACSQDSKQKDTYRSTLILCPPVEKEDTSKEKDQT
ETCDACKQQATSKKKSKSTSKTN-------------SPSSPTTRIGVGATPAHLLSDTMY
TNVANLQQTMLLQQQLFRQALVQNNIDIASK-----------------------------
---------PTTS---------------------FTSPSLSQYQFVSSSQTYTT------
-----------------QPETATLETMMEWKVKRRPDGTRYIARRPV-------------
RNRILKNRAIKISEERAGLTTEDDTVSELKIGRYWTKEERKKHLEKSKERKQRQE--ILL
ASRNIEENGELVIHKPVEKVHSFSSK-----KAANNLDNTVKK--------HKSKRAHKE
GYDNFPTVQEMIVHGPKVP--------TNGKMMGLLSVTTV
>Am-LNX34like
------------------------------------------------------------
------------------------------------------------------------
------------------------------------------------------------
------------------------------------------------------------
------------------------------------------------------------
--MDFVILKVNGQDVSNSSHEDAVRCFQSAQEPIIVEVLRRQPQQQQQQQQQQ-------
-----QHHHG--------------HRVHCA------------------------------
-------------------------QEEKESERREHGTSNAWPNLVSTAVQTDWAGLIET
EEEVVDEQPNDSFED----FLAHDIDFEVNGKD----------VANKEQTENLFAETKNA
VTILVSRCLYQGSPLLSPDHIP--------------SYQNSLIEQLIRQQQQQTEERTKD
SEEDGSTLRPPVPGHTPSSTCSSQASSQKGGGSGGRNGASPARHACD-HERGK------Q
QQWVQEAMKDCSKKIESLCVETEHIYETIPE---SDSEPIYSSPYEHHHR----------
------------------------HHWHHSS------NNHHGPSVVAT--------AQTT
TVSTNASANAINVASSTVIQYTQTGQTVSQTSAGR-WYCSSKSNSSGEEKDSSSAYN---
-TGESNNSSSNNNNSSRDSGRVS-------------SSQYYKDRVTDATGAGNLPADTMY
TNIANLQQTMLLQQQLFKQALDRRNSTIAGS-----------------------------
---------RESSSVTAVAAVPTSAAKKSRSHGNFQAPNLTRYQFVGSQQVCASS-----
-------------NSWIPPEEKGNEVQMEWKVKRRADGTRYIARRPV-------------
RNRILRNRAIKISEERAGHSTEDDTMSEVKIGRYWTKEERKRQLERARERKQRQQEFQLL
QQQQQQQQQQQQQQQQQQQQQQLQQTNELLASEHGEHEKTCKKPLNILELSHKKMARKKN
TLDDFTTVQEMLVHGNRVGGTGGPGGGTGGKLMGLLSVTTV
>Ce-LNX34like
------------------------------------------------------------
```

```
------------------------------------------------------------
------------------------------------------------------------
------------------------------------------------------------
------------------------------------------------------------
-----------MASFTSPDIDEKLAILHREMASLRVECDRLLNKHLIVEHTLS-------
------------------------------------------------------------
----------------------------------QSCSGWPNGAADTSSAYNTGG--ESC
RSVSVTP-----------------------------------------------------
----------------DGVFIPTT-----SNSQMIQSQPSPRVLRRTVKMASPAVQRRLV
PQNLPTSSSSRPPTVLLPHDSPEQPSRPIHR-----------------------------
------------------------------------------------------------
----------------------------------MHQELPIPEDVVS-------------
-MVSSNKTQRSIIRAPSAVFGALKGSSLRKLSISRNIQMTTKVNKEESDRKEKEAEEYAK
RFRIPSYSVVLKEEKRKSSFRIP---------------------VPHYFRSSKAKSKVYQ
VDAPDEEIEEVIDEEKMAATKDLGDTTVHYK-----------------------------
------------------------------------------------------------
-----------------------------WKVKRRCDGSRYIVKRPI-------------
RSQILKKREAQVYRERAPISTDDDAMSELKLGRYHTKEERKRILERENTKKMLKLQQKMM
EKAHPSE-------------QVIYQMS---------QQKLARQ-------------KDAM
VMDEFVTTREVLSSRTRPD-----------GIHGVVSVTTV
>Dm-Slip1
------------------------------------------------------------
------------------------------------------------------------
------------------------------------------------------------
-------------------------------------------MSIADVEYEYVVLKING
YDISHLSRYEAVQKFLQSKETLVVEIRRQKHNALDLELKHGSNAKISKVDNPGELSVLTD
KSAEGTITAASASQQINCPSSTSLKEIET-KTPVVLTLRARSHEDRLGSLQAAS------
---KETQTQSVVGTDVLKDNDLVNTITDNFIEHEHHLFEQCLEPEIDIEEVTLVKG----
----------------------VEQSSSNQIGLIVTSSGIQQSSTDTNKGDILGNVLEHS
EDVFISGVQPESIAYRDGRLRQGDQILRINGLD----------VKNQEELETQIARSSTS
VTLLVSRILYPEDDDDEDIHFEYANTFLPDDYTNVVDKLDKVLLTHVKSLEELSNKSAMQ
SDECYHIPEKNSSDSNVKISSLAKNIIEQSTKSCSKIKLRPNANLDYKKKFNLPLSQEEV
HLQYEYDESEHIYETIPEDSESEPVYCSPYQRSNDKTSIGCSSPIASRPAESLER-----
--------------------TMQQQTQRVAQWLGLKPQYQKTRQTLVG---------RPP
PLKLVQQPTCSRVFTLRSTLTNTSASSSSGVAYSSYGQNNVVTGNAAAPGEEVDNSSSAY
NTGDSNNSASPHQNTTNPDEAIATG--RKLDSTVIDSPNDHLDATGVSTMLLLPFGKSGR
IGLCSSNLPTAYVSERYTNVGSENEIHPLKS-----------------------------
----DIEILRVKPTDDSYSHCPQFNAPNLSSYHFVSSQEVANRCHISTSLQKN-------
--------------ATLLNGESAEEIPMVWKVKRRPDGTRYIVKRPV-------------
RNRPQVALRKNMRYN-EVTTTEDDTISEVKIGRYWTKEERKRHIERAREKRHHQTQQQQQ
------------------------------------------------------------
-----------------------------------------
>Sm-LNX34like
MLEFFKSWTTGFKKNTDQEPVNGQDLRNATHEQAIHAFQTAKEPIIVEVARRDPNVELLQ
KSAPNSNSGP-----------------------------------VNSLDNQNKCSVAIQ
TDPSTAEATLAAMAAAALTTEDIRTALGIRHPGLPTSITFKEENDSIIWDMGFHESENIL
IKEVTLCRKKPDEKFGLTLCYRQGDTSNTSCNVYVGELELNSLAEKSGQIYNGDQILSIN
GNRIKSREHVIDLFQQSQLKVYKRPDQDSGMGRTTDESARTEESSEQEIDNDQHHHHHQY
QQQQRTNFIHGLAQITKLTCENGQFNNNSSGNSLTQDATSLDQKLAFPSDVFS-------
---DYDPIDPIDQELVQLGRLMQSLAVHCRQLVQAKMHYNRIQYPLPIIDNCTSQYGGND
SIVQPQTVLTKVITS------TTTSVINSSSIPTATTTTSSSSSLSSKISNQLTENCYYS
TGNNNLQNTKCIINKSNEGISNGSPLLSTKDNN------------HNMLTRLPRMGTRME
PSIMGSMSSNDSSGSFHDKNLKMSAITTTGKLSHSRFPSNIKDRPVSSERHESQTQQQQQ
TTYCPDSNSKLIEPNSIYPDHLEQCRLNGSGDTSAYCTSEREGITASLSDLGGSLLSLSA
VIPDPHHYHHHRHHSRPQYNSSEVNSTNHQITPNDSFSPSSNCPSNEHKRNMNSTKDLS-
------------------IRSDFRHQHHQHHHPKLHNSNLITEHNPQK--------FVKE
DKSSSPSSIHPNKTKNLQKYSQLHNHPLYPLPPLYCNECYYPIKDISPMKCNTSNINEQL
SIINTSIPSIINKNLHSYEFYDNFYCSLTNKTIMTTNNEKEQSILNQSFKQFNKLINIDK
TNIINEEPTLSDIYETPYASVTIHDEPIGDQYP-----------------------MKCI
SKDPCYPLVYPHPMYTSATCHSKRYQNTAVSASVITGAVSSSSSTAATSTITTTG-----
```

------------MPPSGQVYPMDNWNMMEWVVKKRPDGTRYITRRPISTSTVTTIFADND
SNQLVEERAKRLAEERSGITTDDDAISELKTGRYWNRSERKKQLEKAKADRKRKQLNIIT
QQNSHKN---
--LVLTTTNSSPINKSDGN------------NTTLVTMTTV
>Hm-LNX34
MGIELERFVDSMVHDASCFICSCILEDPLEINPCGHHFCRGCWLNWSTKNSCCAFCGASV
VGVKDSRLLWSLIQN---------------------------------HNVYCIQKEKGC
PAIYKFGFNKLHEGQCLYQGYNRVELNDKCTVCENDSGMTHDCLKELSLKMKQCSIRIIN
LEHENQHLSYKLMNREKEYLERISEIESQFYEESLKYNKEIR------DLRLRAAATQGE
QGKKNNQIYYVHIDLERLNGSLGFNIMGGA----TQDDKHSDGIIVSRIVEGGAADG--K
LQLHDCILEVDSVDLRSATHELAVKAFRESGNPVKMLVRRQISKSATSDFVDVG------
------TQTFGVMFAVTCEEEFNEKHTNSFCKPQETLCKFSNQKRWLNPHEFT-------
-----------------------ESINKEGVSMMSQQNSDTNKNEQYDSAYDTLLTQKSS
RSVRLDTTRNSQVSHDTTRVASPTDSIDKSTTR----------SSPVLKNNDNTSGVTLR
NKIKTEQAENNRLSTFYILPSDIS------AKEWNDVEKVVNFDYDFEYELEEGSEAETT
GKVMVGDQIVQINGCQVKNVENAYKILQENETITLVVARAVYEQQSDCFDEAEETDLDLL
DSINEEAESHYLSLSNPVKLLDNATYDNKAFSYKNHISEKCLHANEKNLGIAPVTN----
--------------------NNNDNNIERLREEIIDNTKSEKMTSVLKN----------F
VRNKFGNSEKTDKRSSCGSDESKDQNKIPRCDSNSSSTSGKSIKTQLNEESAFIKEQKHL
SKMKAYLDSPSIARHRVNQKIIQ--------------PRSSINLETYQAISCRNTKEVDQ
CNLPVKQFSSTYTLPCTENTDKSLNNRRYCDS----------------------------
------ISSLRSSMKTNLVSNKTKPEKHVQIDSHPGWNATEKRVWEEFKIIQS-------
-------------MEAKENSLDPTKIEAEWRVRRSKDGKHVYIKKTNS-----------N
RNKVLKEREDEINKERCGMTTDDDAFTIYQG-QYWDRDQRKRQLVRHQDRRQKLLEKAAI
KATYQNET--------GRKIAEFVQRN---------------------------MTLPGA
VFDNFITIEEILSQRNRSG-----------IFNGPIHVTTI
