# Supplementary material for: Molecular evolution of the LNX gene family
Source: BMC Evol Biol. 2011 Aug 9;11:235. doi: 10.1186/1471-2148-11-235 (PMC3162930; doi:10.1186/1471-2148-11-235)
Supplement: Additional file 3 — Phylogenetic tree of all human PDZ domains. Clustering of related LNX PDZ domains is highlighted in a phylogenetic tree of all human PDZ domains. [file 1471-2148-11-235-S3.PDF]

Additional file 3

Phylogenetic tree of all human PDZ domains

Locations of LNX PDZ domains within the tree are highlighted. PDZ2 domains from all four LNX proteins cluster together, as do the PDZ1 domains. PDZ3 and PDZ4 domains from LNX1 and LNX2 also cluster together. These clusters are distributed quite widely throughout the tree. This pattern is consistent with the early metazoan origins of the LNX domains and with the idea that LNX3/4 proteins evolved from a LNX1/2 like protein through loss of two carboxy-terminal PDZ domains.

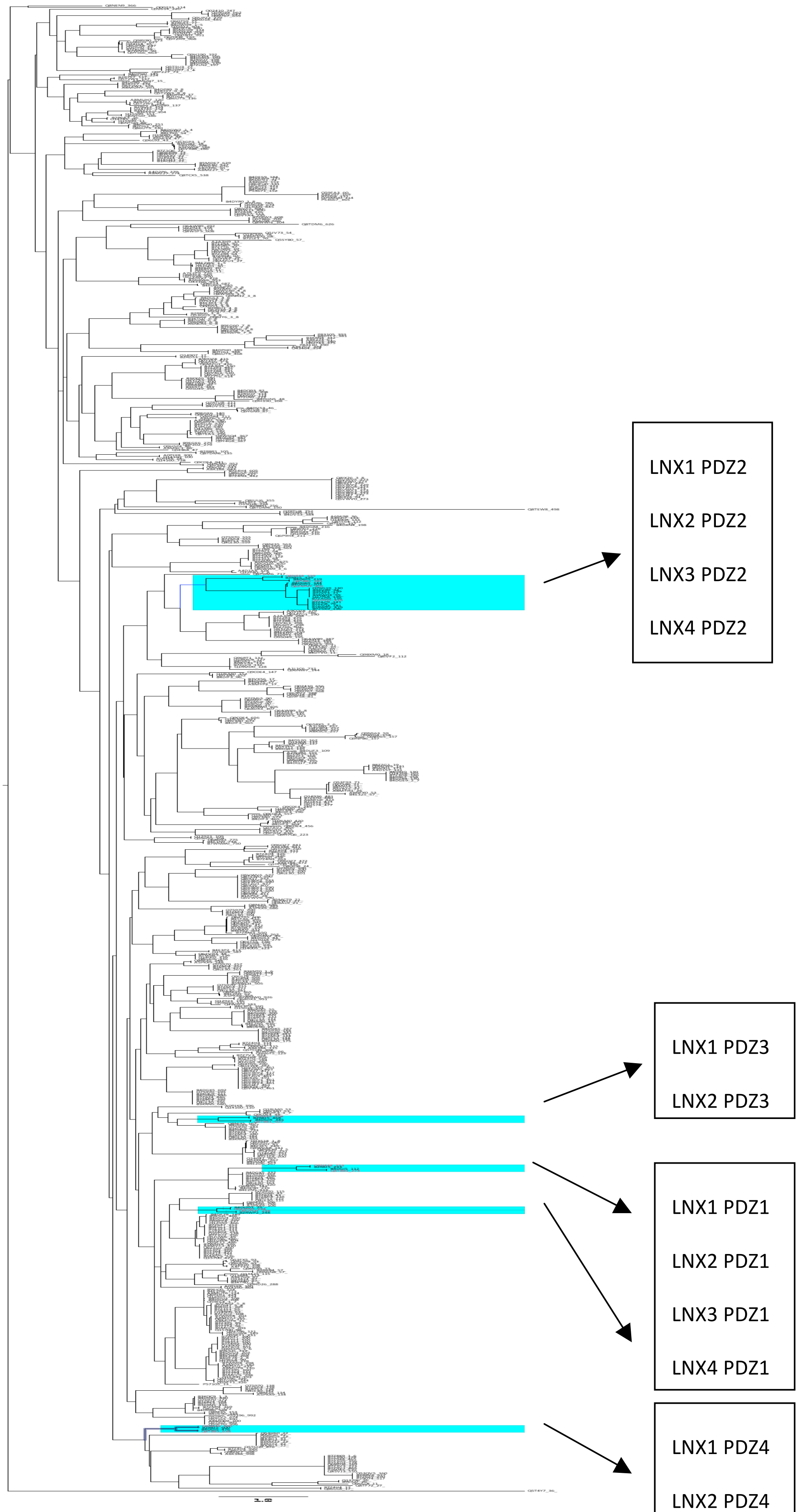
LNX1 PDZ2
LNX2 PDZ2
LNX3 PDZ2
LNX4 PDZ2
LNX1 PDZ3
LNX2 PDZ3
LNX1 PDZ1
LNX2 PDZ1
LNX3 PDZ1
LNX4 PDZ1
LNX1 PDZ4
LNX2 PDZ4
