# Supplementary material for: Molecular evolution of the LNX gene family
Source: BMC Evol Biol. 2011 Aug 9;11:235. doi: 10.1186/1471-2148-11-235 (PMC3162930; doi:10.1186/1471-2148-11-235)
Supplement: Additional file 4 — Conservation of ligand-binding residues in LNX1, LNX2 and MUPP1. Table showing the amino acids identified as determinants of PDZ domain specificity for LNX1, LNX2 and MUPP1. [file 1471-2148-11-235-S4.PDF]

Additional file 4

Conservation of ligand-binding residues in LNX1, LNX2 and MUPP1.

Key amino acids identified as determinants of PDZ domain specificity are shown[1]. Residues at β2-1 and α2-8 determine specificity for the P^0 position of the PDZ ligand, while α2-1, α2-5 contribute to specificity at position P^{-2}. Most residues are conserved between LNX1, LNX2 and MUPP1. Non-conserved residues that may alter binding specificity are highlighted in grey. Nomenclature follows that of Appleton et al (2006)[1].

Reference

[1] Appleton BA, Zhang Y, Wu P, Yin JP, Hunziker W, Skelton NJ, Sidhu SS, Wiesmann C: **Comparative structural analysis of the Erbin PDZ domain and the first PDZ domain of ZO-1. Insights into determinants of PDZ domain specificity**. *J Biol Chem* 2006, **281**(31):22312-22320.

	**LNX1**	**LNX2**	**MUPP1**
**β2-1**			
PDZ1 / 10	Ile	Ile	Leu
PDZ2 / 11	Ile	Ile	Leu
PDZ3 / 12	Met	Met	Ile
PDZ4 / 13	Phe	Phe	Phe
**α2-1**			
PDZ1 / 10	His	His	His
PDZ2 / 11	Phe	Phe	Gln
PDZ3 / 12	Arg	His	His
PDZ4 / 13	His	His	His
**α2-5**			
PDZ1 / 10	Val	Arg	Ile
PDZ2 / 11	Ala	Ala	Ala
PDZ3 / 12	Val	Val	Val
PDZ4 / 13	Ala	Val	Val
**α2-8**			
PDZ1 / 10	Leu	Leu	Leu
PDZ2 / 11	Ile	Ile	Leu
PDZ3 / 12	Leu	Leu	Leu
PDZ4 / 13	Leu	Leu	Leu
