# Supplementary material for: Molecular evolution of the LNX gene family
Source: BMC Evol Biol. 2011 Aug 9;11:235. doi: 10.1186/1471-2148-11-235 (PMC3162930; doi:10.1186/1471-2148-11-235)
Supplement: Additional file 5 — Predicted interactions of LNX1, LNX2, MUPP1 & INADl PDZ domains. Table showing predicted PDZ domain interactions for LNX1 PDZ1-4, LNX2 PDZ1-4, MUPP1 PDZ10-13 and INADl PDZ 8-10. [file 1471-2148-11-235-S5.PDF]

Additional file 5

Predicted interactions of proteins with PDZ domains of human LNX1, LNX2, MUPP1 & INADl. Predicted PDZ domain interactions for LNX1 PDZ1-4, LNX2 PDZ1-4, MUPP1 PDZ10-13 and INADl PDZ 8-10 were obtained from Hui *et al* 2010[1]. Any interaction shared between LNX1/LNX2 and MUPP1/INADl was extracted from this dataset. PDZ domains predicted to interact with the proteins indicated in the left column are indicated by a number and color coded to highlight the phylogenetically related domains as follows.

LNX1 PDZ1 <=> LNX2 PDZ1 <=> MUPP1 PDZ10 <=> INADl PDZ8

LNX1 PDZ2 <=> LNX2 PDZ2 <=> MUPP1 PDZ11 <=> INADl PDZ9

LNX1 PDZ3 <=> LNX2 PDZ3 <=> MUPP1 PDZ12 <=> INADl PDZ10

LNX1 PDZ4 <=> LNX2 PDZ4 <=> MUPP1 PDZ13

Many of the predicted interactions are mediated by the phylogenetically related domains in LNX1/2 and MUPP1/INADl. Beyond that there is a relationship between the first two domains in LNX1/LNX2 and the equivalent two domains in MUPP1/INADl and likewise between the two carboxy terminal domains of LNX1/LNX2 and carboxy terminal domains in MUPP1/INADl. Thus almost all interaction partners predicted for PDZ1 or PDZ2 of LNX1/LNX2 interact with either PDZ10 or PDZ11 of MUPP1, or PDZ8 or PDZ9 of INADl (the exception is KALRN). Likewise almost all interaction partners predicted for PDZ3 or PDZ4 of LNX1/LNX2 interact with either PDZ12 or PDZ13 of MUPP1, or PDZ10 of INADl (the exception is C11orf10). The similar binding specificity of these pairs of domains in LNX and MUPP1 suggest that in the common ancestor of these proteins these pairs of domains were closely related and that each pair might have arisen from duplication of a single domain.

Reference

1. Hui S, Bader GD: **Proteome scanning to predict PDZ domain interactions using support vector machines**. *BMC Bioinformatics* 2010, **11**:507.

Protein	*LNX1*	*LNX2*	*MUPP1*	*INADl*
**SSTR3**	1	-	10	8
**TRAPPC8**	1	-	10	8
**HTR2C**	1	-	10	8
**MKI67IP**	1	-	10	8
**PDGFA**	1	-	10	8
**MYO5A**	1	-	10	8
**CLDN9**	1	-	10	8
**LAMA5**	-	1	10	8,9
**KDM1B**	-	1,2	10	8
**MYH8**	-	1	10	8
**EPHA7**	2	2	11	9
**SMCR7L**	2	2	11	9
**SSBP1**	2	2	11	9
**MMP27**	-	2	11	9
**SOD1**	2	-	11	9
**CNKSR2**	-	3, 4	13	10
**KALRN**	1,3	3, 4	-	10
**SLC15A5**	-	3, 4	-	10
**SLC22A5**	-	3	-	10
**TMEM215**	-	3, 4	13	-
**C3orf70**	-	3, 4	13	-
**GPR125**	-	4	13	-
**NET1**	-	4	13	-
**IL22RA2**	-	3, 4	13	-
**MCC**	-	4	13	-
**GH1**	2	2	-	8
**PLEKHM1**	2	2	10	-
**PRKCA**	2	-	10	8
**KREMEN2**	2	2	10	8
**NR4A2**	2	2	10	8
**TBL1XR1**	2	2	10	8
**DCC**	2	2	-	8
**CDYL**	2	2	-	8

Protein	*LNX1*	*LNX2*	*MUPP1*	*INADl*
**SLC25A26**	2	-	-	8
**RASSF8**	2	2	-	9
**PPAPDC3**	2	-	10	8
**NEK6**	2	2	10	8
**GRB10**	2	-	10	8
**DGKI**	2	-	13	-
**ZNF655**	2	-	-	8
**KIF3C**	2	-	10	8
**TOMM5**	2	-	10	8
**C11orf10**	2, 3	2	-	9
**ARPC4**	2	-	10	8
**ANGEL2**	2	-	10	8
**GZMA**	2	-	-	8
**RHBDL3**	-	3	13	-
**NUP62CL**	-	3	-	10
**ABCC4**	-	3, 4	13	10
**SHKBP1**	-	3	13	-
**PHACTR2**	-	3	13	-
**ARHGAP6**	-	3	13	-
**TMEM200A**	-	3	13	-
**TRPV3**	-	3	13	-
**SLC16A3**	-	3	13	-
**TMIGD1**	-	4	-	10
**SLC6A11**	-	3	13	-
**C1orf159**	1	-	10	8
**KCNJ10**	1	-	10	8
**HEATR1**	-	2	-	8
**IFT27**	-	3	13	-
**NUP37**	-	3	13	-
