# Supplementary material for: Molecular evolution of the LNX gene family
Source: BMC Evol Biol. 2011 Aug 9;11:235. doi: 10.1186/1471-2148-11-235 (PMC3162930; doi:10.1186/1471-2148-11-235)
Supplement: Additional file 6 — Genbank accession numbers. Table showing Genbank accession numbers for LNX and MUPP1 homologs referred to in this study. [file 1471-2148-11-235-S6.PDF]

Additional file 6

Genbank accession numbers for LNX and MUPP1 homologs referred to in this study

LNX homologs

**Species**	**LNX1/LNX2/LNX2b**	**LNX3/LNX4/LNX5**
Trichoplax adhaerens	-	XP_002111914.1
Hydra magnipapillata	-	XP_002159074
Schistosoma mansoni	XP_002576205	XP_002574957
Branchiostoma floridae	XP_002588339.1	XP_002601701
Saccoglossus kowalevskii	XP_002738713	XP_002741954.1
Strongylocentrotus pupuratus	XP_001186128	XP_780965.2
Ciona intestinalis	-	XP_002125622
Tribolium castaneum	-	XP_966550
Apis mellifera	-	XP_393616
Caenorhabditis elegans	-	NP_501262
Drosophila melanogaster	-	NP_651933.1
Homo sapiens	NP_001119800.1 (LNX1)	NP_055824.1 (LNX3)
	NP_699202.1 (LNX2)	NP_001158067.1 (LNX4)
		NP_115901.2 (LNX5)
Mus musculus	NP_001153049.1 (LNX1)	NP_061372.2 (LNX3)
	NP_542985.4 (LNX2)	NP_001158065.1 (LNX4)
		NP_001025039.1 (LNX5)
Gallus gallus	XP_001232234.1 (LNX1)	XP_414432.2 (LNX3)
	XP_417122.2 (LNX2)	XP_416030.2 (LNX4)
	XP_420296.1 (LNX2b)	
Xenopus tropicalis	XP_002934714.1 (LNX1)	XP_002938429.1 (LNX3)
	NP_001072606.1 (LNX2a)	XP_002932856.1 (LNX4)
	XP_002931874.1 (LNX2b)	NP_001135536.1 (LNX5)
Danio rerio	NP_001068581.1 (LNX1)	XP_001344551.1 (LNX3)
	NP_001106696.2 (LNX2a)	NP_001076392.1 (LNX4)
	NP_998105.2 (LNX2b)	XP_001341731.3 (LNX5)

MUPP1 homologs

**Species**	
Homo sapiens	NP_003820.2
Trichoplax adhaerens	XP_002113893.1
Monosiga brevicollis	XP_001745891.1
